# Enterotype-based Analysis of Gut Microbiota along the Conventional Adenoma-Carcinoma Colorectal Cancer Pathway

**DOI:** 10.1038/s41598-019-45588-z

**Published:** 2019-07-29

**Authors:** Tzu-Wei Yang, Wei-Hsiang Lee, Siang-Jyun Tu, Wei-Chih Huang, Hui-Mei Chen, Ting-Hsuan Sun, Ming-Chang Tsai, Chi-Chih Wang, Hsuan-Yi Chen, Chi-Chou Huang, Bei-Hao Shiu, Tzu-Ling Yang, Hsin-Tzu Huang, Yu-Pao Chou, Chih-Hung Chou, Ya-Rong Huang, Yi-Run Sun, Chao Liang, Feng-Mao Lin, Shinn-Ying Ho, Wen-Liang Chen, Shun-Fa Yang, Kwo-Chang Ueng, Hsien-Da Huang, Chien-Ning Huang, Yuh-Jyh Jong, Chun-Che Lin

**Affiliations:** 1 Division of Gastroenterology and Hepatology, Department of Internal Medicine, Chung Shan Medical University Hospital, Taichung, 402 Taiwan; 2 School of Medicine, Chung Shan Medical University, Taichung, 402 Taiwan; 3 Institute and Department of Biological Science and Technology, College of Biological Science and Technology, National Chiao Tung University, Hsinchu, 300 Taiwan; 4 Institute of Bioinformatics and Systems Biology, College of Biological Science and Technology, National Chiao Tung University, Hsinchu, 300 Taiwan; 5 Department of Medical Research, Chung Shan Medical University Hospital, Taichung, 402 Taiwan; 6 Institute of Medicine, Chung Shan Medical University, Taichung, 402 Taiwan; 7 Division of Colon and Rectum, Department of Surgery, Chung Shan Medical University Hospital, Taichung, 402 Taiwan; 8 Division of Endocrinology and Metabolism, Department of Internal Medicine, Chung Shan Medical University Hospital, Taichung, 402 Taiwan; 9 Graduate Institute of Clinical Medicine, College of Medicine, Kaohsiung Medical University, Kaohsiung, 807 Taiwan; 10 Departments of Pediatrics and Laboratory Medicine, Kaohsiung Medical University Hospital, Kaohsiung Medical University, Kaohsiung, 807 Taiwan; 11 Institute of Molecular Medicine and Bioengineering, College of Biological Science and Technology, National Chiao Tung University, Hsinchu, 300 Taiwan; 12 Warshel Institute For Computational Biology, The Chinese University of Hong Kong, Shenzhen, 518172, Longgang District, Shenzhen, China; 13 School of Life and Health Sciences, The Chinese University of Hong Kong, Shenzhen, 518172, Longgang District, Shenzhen, China; 14 School of Sciences and Engineering, The Chinese University of Hong Kong, Shenzhen, 518172, Longgang District, Shenzhen, China

**Keywords:** Colon cancer, Colon cancer

## Abstract

The dysbiosis of human gut microbiota is strongly associated with the development of colorectal cancer (CRC). The dysbiotic features of the transition from advanced polyp to early-stage CRC are largely unknown. We performed a 16S rRNA gene sequencing and enterotype-based gut microbiota analysis study. In addition to *Bacteroides*- and *Prevotella*-dominated enterotypes, we identified an *Escherichia*-dominated enterotype. We found that the dysbiotic features of CRC were dissimilar in overall samples and especially *Escherichia*-dominated enterotype. Besides a higher abundance of *Fusobacterium*, *Enterococcus*, and *Aeromonas* in all CRC faecal microbiota, we found that the most notable characteristic of CRC faecal microbiota was a decreased abundance of potential beneficial butyrate-producing bacteria. Notably, *Oscillospira* was depleted in the transition from advanced adenoma to stage 0 CRC, whereas *Haemophilus* was depleted in the transition from stage 0 to early-stage CRC. We further identified 7 different CAGs by analysing bacterial clusters. The abundance of microbiota in cluster 3 significantly increased in the CRC group, whereas that of cluster 5 decreased. The abundance of both cluster 5 and cluster 7 decreased in the *Escherichia*-dominated enterotype of the CRC group. We present the first enterotype-based faecal microbiota analysis. The gut microbiota of colorectal neoplasms can be influenced by its enterotype.

## Introduction

A trend towards a decreased overall incidence (a decrease of 3.3% per year in men and 3.0% in women) of and mortality (a decrease of 2.5% per year in men and 3.0% in women) from colorectal cancer (CRC) was noted from 2006 to 2010^1^ and was attributed to the use of screening tests to detect colon neoplasms at early time points and the removal of pre-malignant lesions^2–4^. Nonetheless, CRC was still the fourth most common cause of cancer-related deaths worldwide in 2012^5^ and was the third most common cancer in the United States in 2014^1^. Despite the availability of various methods to screen for CRC, approximately 30% of the adults in the US do not receive appropriate screenings for their age. Colonoscopy is the gold standard for the accurate diagnosis of CRC^6,7^. However, the invasive and unpleasant nature of colonoscopies often causes patients unwanted pain and discomfort, leading more than half to prefer non-invasive screening methods^6,8^. Current “non-invasive” faecal screening tests, including the faecal immunochemical (FIT) and the multi-target faecal DNA tests, have significantly improved the detection rate of CRC^9,10^. However, their ability to detect pre-cancerous or small lesions is limited.

Contributors to the pathogenesis of CRC include chronic inflammation and the accumulation of genetic, epigenetic, diet, and environmental factors^11,12^. As the well-described carcinogenic potential of infectious agents contributes to more than 18% of the global cancer burden (e.g., gastric cancer, which can be caused by *Helicobacter pylori*)^13^, emerging evidence suggests that a dysbiosis of human gut microbiota is associated with CRC^13–16^. It has been hypothesized that certain pathogens interact with the colon epithelium by influencing the host’s immune system, increasing its mutagenic potential through chronic inflammation, possessing bacteria-derived virulence factors, and creating DNA-damaging and non-DNA-damaging metabolites^17^. For example, *Fusobacterium nucleatum (Fn)* is prevalent in CRC and pre-malignant colorectal lesions^18,19^ and has been associated with a poor prognosis^20^. Alternations in the composition of the gut microbiome have also been observed along the adenoma-carcinoma sequence^16^. An altered microenvironment that leads to a different gut microbe composition is thought to be a biomarker that can differentiate healthy subjects from those with colonic neoplasms^16,21,22^. To analyse these specific dysbiotic features, the human faecal microbiome may be a new detection tool for CRC^14,16,21,22^. Furthermore, manipulating the gut microbiome may affect the progression of colonic neoplasms.

However, previous studies that used differing clustering and grouping strategies produced heterogeneous results^14–16,21,22^. Given that the human gut microbiome can be characterized by changes in the level of one of three robust genera—*Bacteroides*, *Prevotella*, and *Ruminococcus*—these categories have been defined as “enterotypes”^23^. Enterotypes are stable and are strongly associated with long-term diets; a protein and animal fat-rich diet has been associated with the *Bacteroides*-dominated enterotype, while a carbohydrate-rich diet has been linked to the *Prevotella*-dominated enterotype^24^. We hypothesized that changes in the gut microbiome in patients with colorectal neoplasms are different among enterotypes.

Here, we systemically investigated the microbial composition of human stool samples at various points along the conventional adenoma to carcinoma sequence using enterotype-based and co-abundance group (CAG) analysis.

## Results

### Sample collection and NGS OTU mapping

We analysed stool samples from 283 individuals, including 104 from normal controls, 117 from patients with adenomatous polyps, and 62 from patients with CRC (Table 1). One-hundred seventy-three of the subjects were males, and 110 were females. Their ages ranged from 40 to 86, with a mean of 60.96 **±** 10.11 years old. We generated 13,671,987 quality-filtered sequence reads, with 48,311 average reads per sample. Sequence reads were mapped to the bacteria in the SILVA database. We mapped all sequences into 277 genera. The most dominant bacterial phyla were Bacteroidetes, Proteobacteria, and Firmicutes, which covered more than 95% of our sequenced reads. These phyla were present in all individuals, with minor variations between groups (Table S1).

**Table 1:** Summary of Characteristics of Subjects Enrolled.

Characteristics		Total	Normal	Adenomatous polyp		Colorectal cancer		Late stage	p-value*
				Small adenoma	Advanced adenoma	0	Early stage		
							I-II	III-IV	
Total subjects		283	104	58	59	21	21	20	
Gender (M:F)		173:110	53:51	40:18	40:19	14:7	15:5	11:10	0.025
Age (mean,SD)		60.96 ± 10.11	60.71 ± 10.44	60.96 ± 10.09	61.12 ± 10.10	61.00 ± 10.07	61.08 ± 10.14	61.09 ± 10.27	
BMI (mean,SD)		24.08 ± 3.42	23.67 ± 3.34	24.08 ± 3.42	24.12 ± 3.44	24.11 ± 3.43	24.09 ± 3.43	24.01 ± 3.43	
**Underlying disease**									
Hypertension	Yes	120	35	24	33	7	10	11	0.03
	No	145	65	29	20	12	10	9	
	Unknown	18	4	5	6	2	1	0	
Hyperlipidemia	Yes	112	35	26	30	10	7	4	0.6
	No	139	59	25	20	10	10	15	
	Unknown	32	10	7	9	1	4	1	
Diabetes mellitus	Yes	60	19	7	15	9	6	4	0.29
	No	201	75	48	38	11	13	16	
	Unknown	22	10	3	6	1	2	0	
Cardiovascular disease	Yes	50	16	7	13	3	9	2	0.645
	No	199	73	48	35	16	9	18	
	Unknown	34	15	3	11	2	3	0	
**Family history**									
Colon polyp	Yes	7	4	1	1	0	1	0	0.763
	No	276	100	57	58	21	20	20	
Colorectal cancer	Yes	27	7	6	8	2	4	0	0.269
	No	256	97	52	51	19	17	20	
**Life style**									
Smoking	Current smoker	53	13	9	14	5	6	6	0.013
	Ex-smoker	73	20	21	14	7	6	5	
	Non-smoker	157	71	28	31	9	9	9	
Lesion site	Proximal	82	NA	29	28	7	10	8	0.283
	Distal	98	NA	29	31	14	11	12	

*p-value was performed with 3 groups (Normal, Adenoma, Cancer) by chi-square test.

### Enterotypes and biodiversity analysis

**Figure 1:**
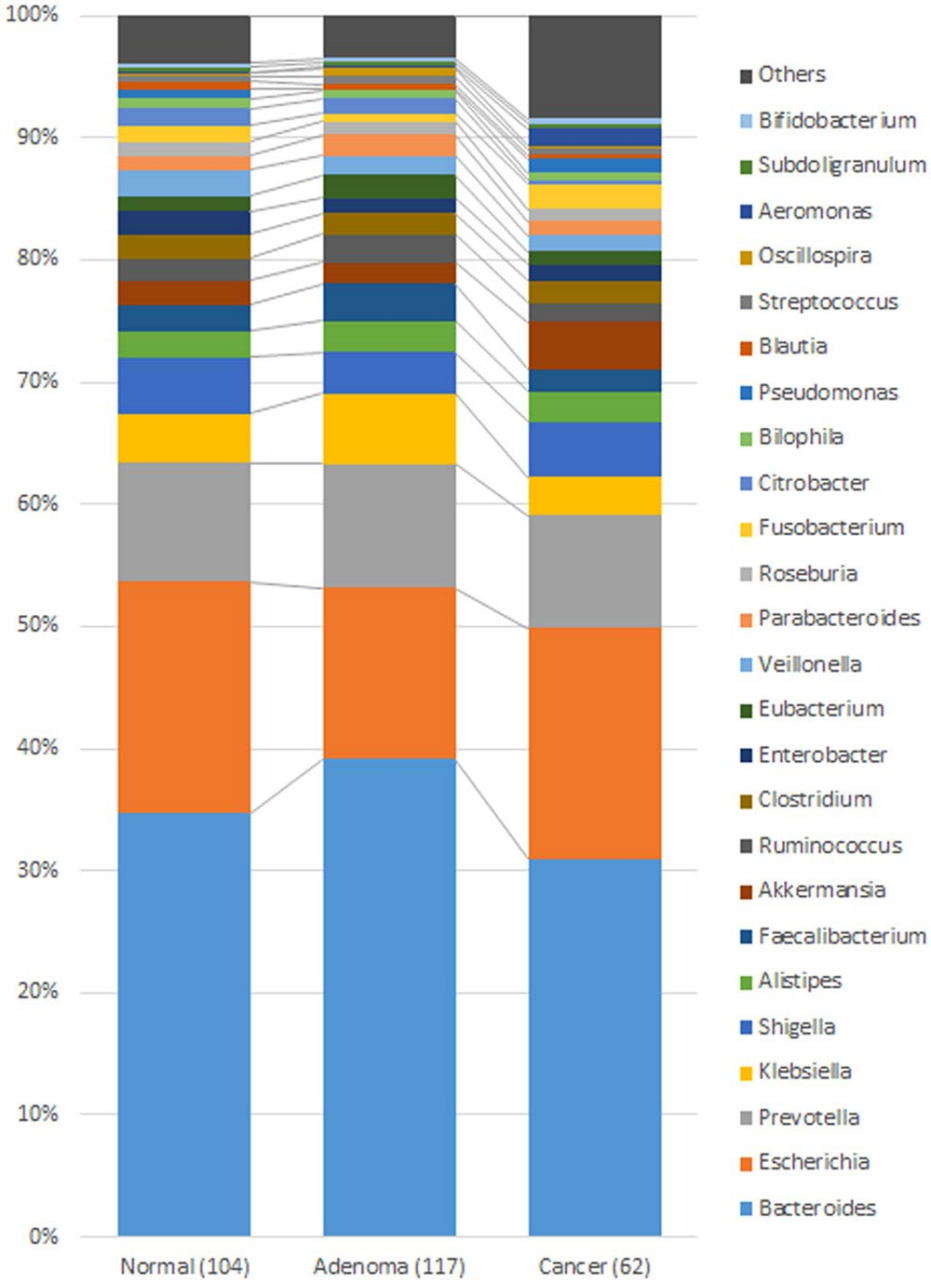
Overall microbial flora at the genus level The average of the top 25 genera of each group, which occupied more than 90% of the relative abundance of the three groups. These groups shared the same top 16 genera, arranged in a slightly different order.

**Figure 2:**
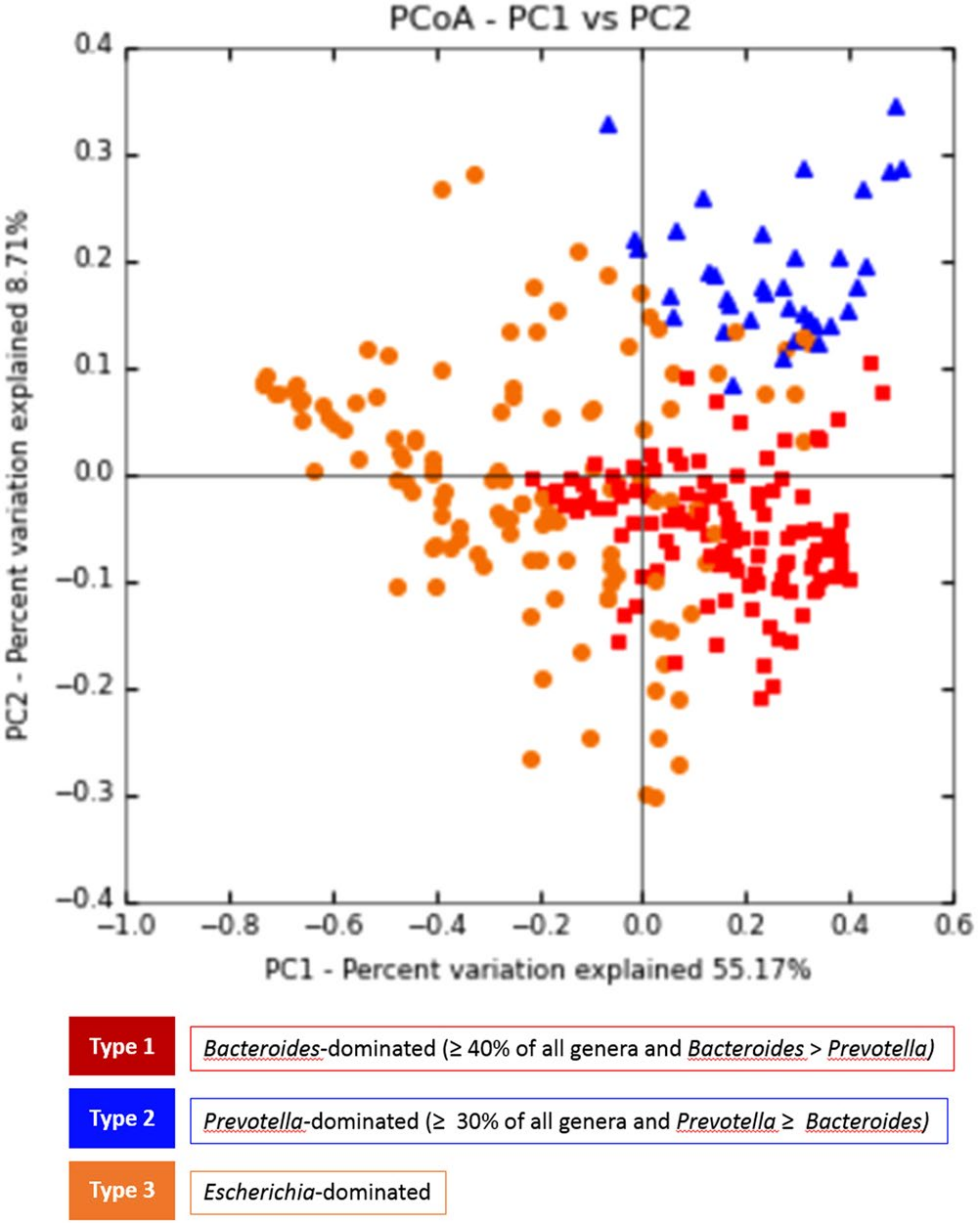
PCoA plot of enterotypes using weighted PCoA Enterotypes were defined as microbial flora dominated by genus *Bacteroides*, *Prevotella*, or *Escherichia*. Samples with a relative abundance of *Bacteroides* over 40% with levels greater than *Prevotella* were assigned to enterotype 1. Samples with a relative abundance of *Prevotella* of over 30% with levels greater than or equal to *Bacteroides* were assigned *to* enterotype 2. All others were assigned to enterotype 3, which was found to be dominated by *Escherichia*.

At the genus level, *Bacteroides*, *Escherichia*, and *Prevotella* contributed to the majority of the human gut microbiota, with an average prevalence of 36.52%, 16.03%, and 9.84%, respectively (Fig. 1, Table S2 and Fig. S2A). The weighted principal coordinates analysis (PCoA) of all stool samples demonstrated strong clustering into three enterotypes that were dominated by the 3 genera: enterotype 1 contained a high proportion of *Bacteroides* (≥40% of all genera, with more *Bacteroides* than *Prevotella*); enterotype 2 contained a high proportion of *Prevotella* (≥30% of all genera, with more *Prevotella* than *Bacteroides*); and enterotype 3 contained a higher proportion of *Escherichia* mixed with other genera (Figs 2 and S2B–D). The PCoA plot represents the microbiota of all faecal samples, which were significantly different and clearly separated into the 3 enterotypes. However, there was no difference in the incidence of colorectal neoplasms between enterotypes, although enterotypes 1 and 3 contained most of the cases (Supplementary Table S3).

**Figure 3:**
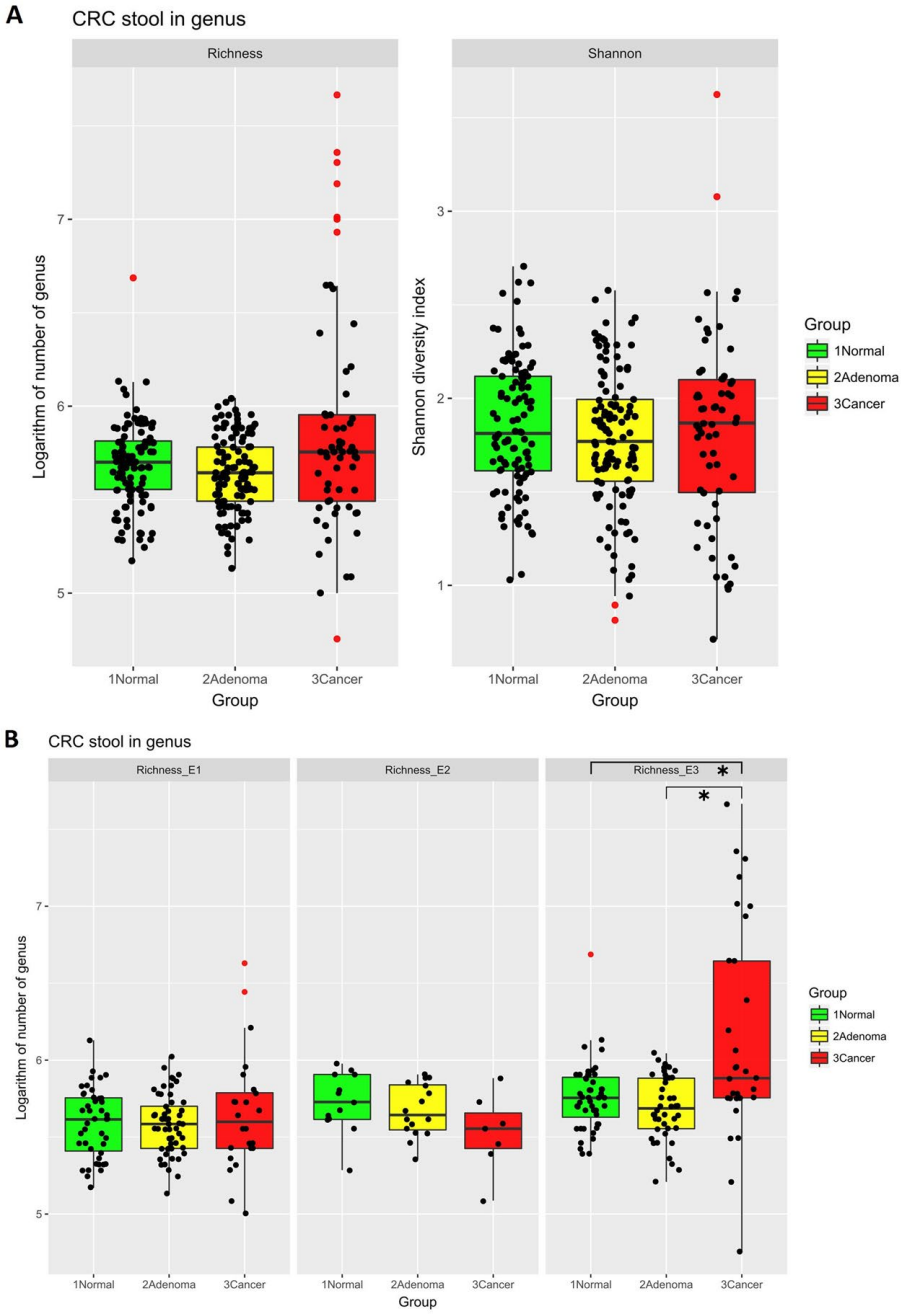
**(A)** Richness and Shannon diversity indices by group in all faecal samples The Shannon diversity index and the binary logarithm of the genus richness of each sample were calculated in all three groups. Each group had a similar richness and Shannon diversity index, with only the cancer group having a slightly higher variation in richness that was not significant. Each dot represents one sample, and outlier samples are marked as red dots. (**B)** Richness index by group across enterotypes The binary logarithm of the genus richness of each sample in the three enterotypes. Enterotype 1 and 2 had a similar richness in each group. In enterotype 3, the cancer group had a significantly higher and more varied richness. (*p < 0.01).

We then calculated the richness and Shannon diversity index between the normal, adenoma, and CRC groups, which were not significantly different between groups (Fig. 3A). When we took the enterotype into consideration, CRC group members that are in enterotype 3 are significantly richer than their adenoma and normal counterparts in the same enterotype (*p* < 0.01, Fig. 3B). However, the Shannon diversity and richness measurements were not different between the groups within the 3 enterotypes. Subgroup analysis also showed a trend towards increasing richness from stage 0 to late-stage CRC, although the Shannon diversity index remained equivocal (Supplementary Fig. S1A–C).

### Faecal microbiota differs between the CRC, adenoma and normal control groups in different enterotypes

**Figure 4:**
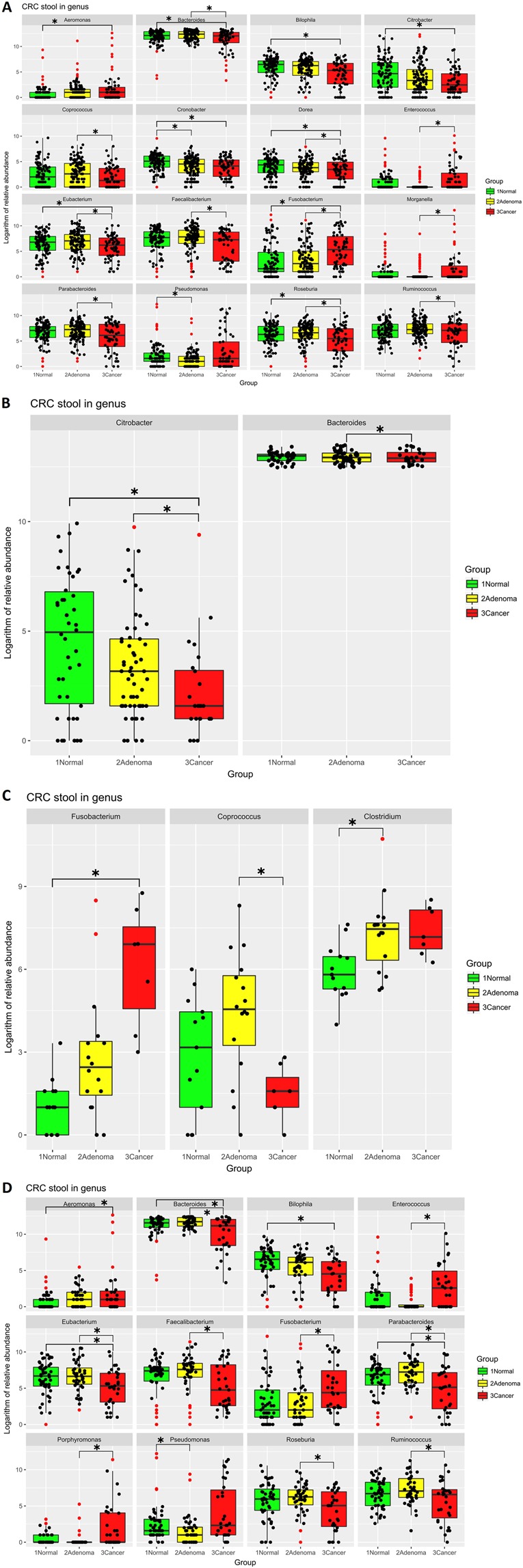
**(A)** Relative overall sample abundance in the 3 groups Binary logarithms of the relative abundance of a single genus in the normal, adenoma, and cancer groups. Each genus was present in more than 50% of the samples in the cancer group. Most of the significant differences were between the cancer group and the other 2 groups. (**B)** Relative abundance between the 3 groups in enterotype I The binary logarithm of the relative abundance of a single genus in enterotype I. Both genera were present in more than 50% of the cancer group samples. The significance observed in the *Citrobacter* levels was between the cancer group and the other 2 groups, while *Bacteroides* levels were significantly different between cancer and adenoma groups. (**C**) Relative abundance between the 3 groups in enterotype II A binary logarithm of the relative abundance of a single genus in enterotype II. All genera were present in more than 50% of the cancer group samples. *Fusobacterium* levels were significantly different between cancer and normal groups, *Coprococcus* levels were different between cancer and adenoma groups, and *Clostridium* differences were between the normal and adenoma groups. (**D**) The relative abundance between 3 groups in enterotype III Binary logarithm of relative abundance of single genus in enterotype III, and all genera present in more than 50% of samples in cancer group. Most of the significances are between cancer group and other 2 groups. Each dot represents one sample, and outlier samples are marked as red dots. (*p < 0.01).

Overall, the abundance of sixteen of the genera was significantly different between the normal control, adenoma, and CRC groups. In particular, the relative abundance of *Fusobacterium*, *Enterococcus*, and *Morganella* was significantly greater in CRC patients relative to those with adenomas (all *p* < 0.01, Fig. 4A, Table S4A).

Given that the enterotype was influenced by long-term diet, we assumed that dysbiotic features might be different within enterotypes. Further, we analysed the abundance of different groups within the 3 enterotypes. In enterotype 1, *Bacteroides* and *Citrobacter* were less common in individuals with CRC. In enterotype II, *Fusobacterium* was more abundant in individuals with CRC, while the *Coprococcus* levels were lower. The abundance of twelve genera were significantly different in enterotype III. In particular, we observed an overexpression of pathogenic bacteria, including *Aeromonas*, *Enterococcus*, *Fusobacterium*, and *Porphyromonas* (all *p* < 0.01, Fig. 4B–D, Supplementary Table S4B–D).

### “Key bacteria” in the transition from a pre-cancerous polyp to CRC

**Figure 5:**
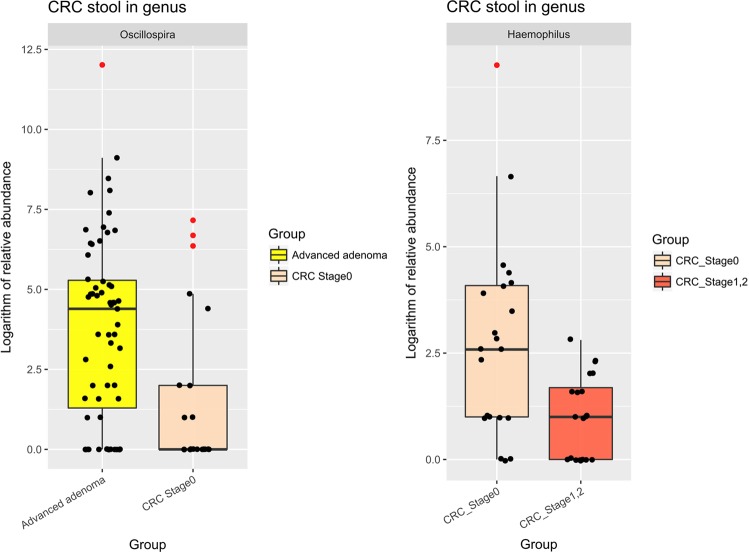
Relative abundance in the transition from an advanced adenoma to stage 0 CRC to early-stage CRC A binary logarithm of the relative abundance of a single genus between the two sub-groups. The relative abundance of *Oscillospira* in the CRC stage 0 group was significantly lower than in the advanced adenoma group and was also present in approximately 35% fewer samples in the CRC stage 0 group. The relative abundance of *Haemophilus* in the CRC stage 0 group was significantly higher than in the CRC stage 1, 2 group. Each dot represents one sample, and outlier samples are marked as red dots. (*p < 0.01).

We further performed a subgroup analysis to observe changes in the abundance of “key bacteria” during the transition from an advanced polyp to early-stage (stage 0, 1, or 2) CRC. We found a significantly decreased abundance of four butyrate-producing bacteria during this progression: *Eubacterium*, *Roseburia*, *Faecalibacterium*, and *Oscillospira* (all *p* < 0.01, Fig. S3). Of note, less *Oscillospira* was found in stage 0 CRC relative to advanced polyps, while reduced *Haemophilus* was observed in stage 1 and 2 CRC relative to stage 0 CRC (all *p* < 0.01, Fig. 5).

### CRC correlation clustering and classifiers for CRC

**Figure 6:**
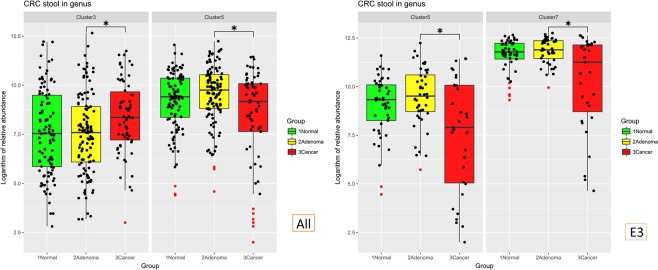
The relative abundance of clustered CAGs, with significant differences between groups Normal and cancer samples were selected for Pearson’s correlation analysis, and 7 CAGs were combined. The abundance of cluster 3 was significantly increased in the CRC group, whereas cluster 5 was decreased. In enterotype 3 (the *Escherichia*-predominated enterotype), the CRC group had decreased levels of clusters 5 and 7.

We performed a Spearman’s correlation analysis to identify CAGs between the normal, adenomatous polyp and CRC groups. Only the genera whose appearance were greater than 50% in cancer group were selected. Using different combinations of samples from the normal (N), adenomatous polyp (A), and CRC (C) groups (Supplementary Figs S4–S7 and Tables S5–S12), we found that the CAG created from the combination of N and C faecal samples had the best ability to classify adenoma and CRC samples (Table S6, Fig. 6). The abundance of cluster 3 significantly increased in the CRC group, whereas that of cluster 5 decreased. In enterotype 3 (*Escherichia*-predominated enterotype), the CRC group contained decreased levels of clusters 5 and 7 (Fig. 6).

### Network of bacteria

**Figure 7:**
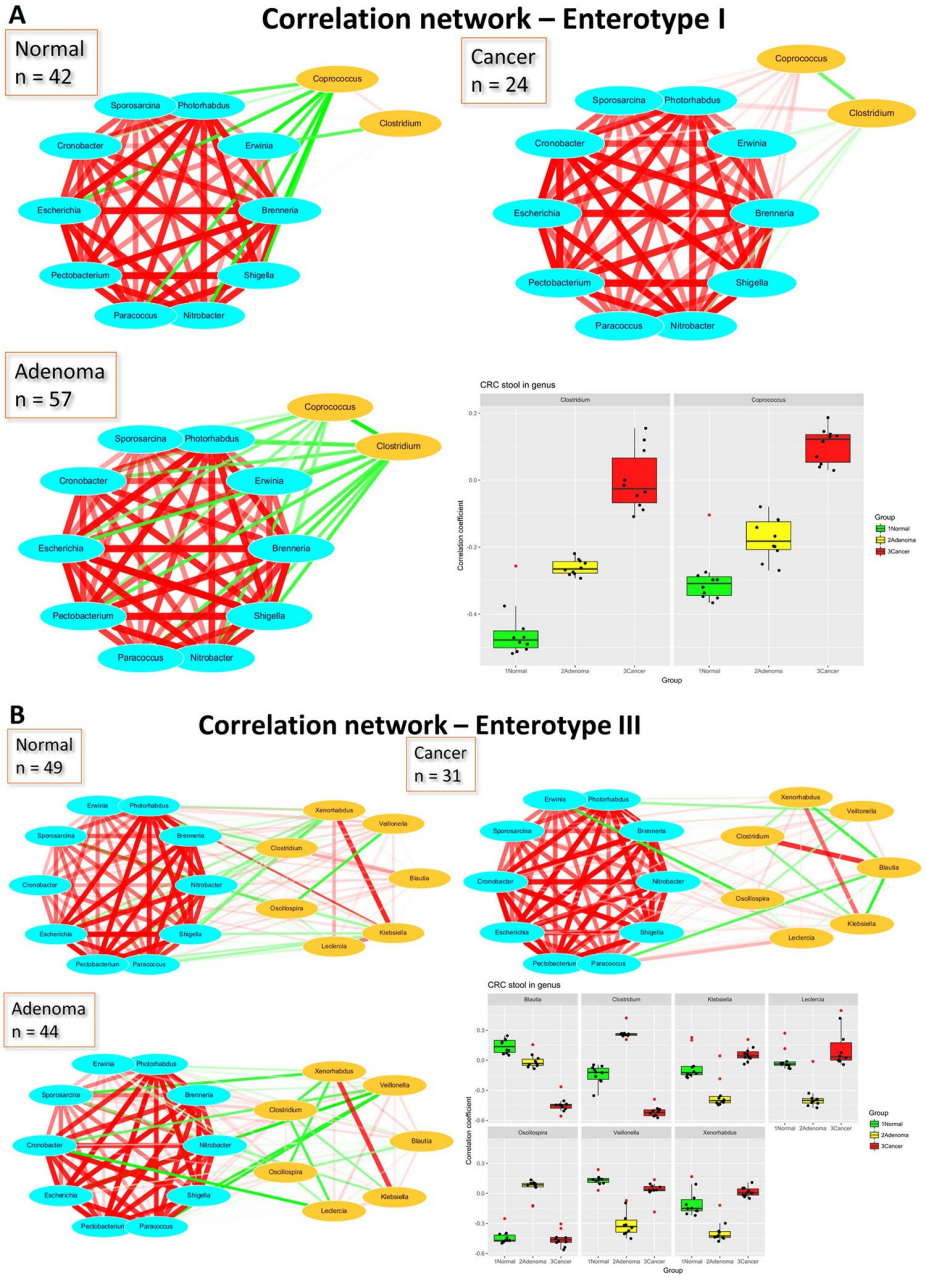
(**A)** Network analysis of stool microbiota using Pearson’s correlation coefficients (Enterotype I) Correlation coefficients between 10 genera of CAG 2, *Clostridium* and *Coprococcus*. **(B**) Enterotype III Correlation coefficients network between 10 genera of CAG and 2 and 7 other genera.

The composition of each CAG varied between different sample combinations. However, cluster 2, which contained 10 genera—*Brenneria*, *Cronobacter*, *Erwinia*, *Escherichia*, *Nitrobacter*, *Paracoccus*, *Pectobacterium*, *Photorhabdus*, *Shigella*, and *Sporosarcina*—stayed the same in all groups. Furthermore, the genera also clustered together in all enterotypes. These 10 genera clustered in every group, with a high correlation coefficient (Table S13, in the cancer group). This steady CAG provided a satisfactory standard for identifying specific genera that differed between groups. We identified two genera, *Clostridium* and *Coprococcus*, whose correlation coefficient with this CAG varied between the normal, adenoma, and cancer groups in enterotype 1 (Figs 7A and S8A). In enterotype 2, 9 genera, including *Citrobacter*, *Clostridium*, *Fusobacterium*, *Klebsiella*, *Lactobacillus*, *Leclercia*, *Peptostreptococcus*, *Synergistes*, and *Veillonella*, had a high variation between groups in their correlation with this CAG (Figs S8B and S9). We also found that the *Blautia*, *Clostridium*, *Klebsiella*, *Leclercia*, *Oscillospira*, *Veillonella*, and *Xenorhabdus* genera had substantially different correlation coefficients within this CAG in the normal, adenoma, and cancer groups in enterotype 3 (Figs 7B and S8C).

## Discussion

The study confirmed that faecal microbiota differ along the adenoma-to-carcinoma sequence and across enterotypes. A previous metagenome-wide association study reported a greater prevalence of CRC faecal microbiota, suggesting an overgrowth of potential pathogenic taxa^16^. Identical findings were observed in the present study in CRC from enterotype 3 and in late-stage CRC. The increased abundance of *Fusobacterium*, *Enterococcus*, and *Aeromonas* in the CRC group was consistent with previous reports^14,15,25^. The high abundance of *Porphyromonas that was* previously reported was observed only in enterotype 3^14,15^. Beyond *Bacteroides*-dominated and *Prevotella*-dominated enterotypes, we observed an *Escherichia*-dominated enterotype 3 in the Taiwanese population that was different from the *Ruminococcus* enterotype^26^.

Consensus is that no single bacteria is representative of the dysbiosis of CRC and that increased levels of potentially pathogenic bacteria are not the only biomarkers of CRC^15,16,27^. The loss of potentially beneficial taxa may be more predictive of colorectal neoplasms^27^. In this study, we identified that a decreased abundance of CAG cluster 5 and cluster 7, composed primarily of butyrate-producing bacteria, is a suitable marker of CRC. In previous study of Flemer, B. *et al*.^15^, they mentioned that “no single OTU tested being increased in all individuals with CRC” and “community structure can be more informative than abundance differences of individual taxa”. Although we identified several significant genera in different enterotypes, not a single genus showed significance in all groups. Here, we not only tried to identify significant markers in groups, but also found a highly correlated group of 10 genera—*Brenneria*, *Cronobacter*, *Erwinia*, *Escherichia*, *Nitrobacter*, *Paracoccus*, *Pectobacterium*, *Photorhabdus*, *Shigella*, and *Sporosarcina*—stayed the same in all groups and enterotypes. This might suggest a least part of the gut bacteria function as groups.

Clinically, CRCs occurred in patients with relatively healthy diets or in vegetarians, who were considered to be at a decreased risk of CRC^28,29^. Genetic and environmental factors may play a role in this situation. Given that enterotypes are associated with long-term diet, we assumed that the “key bacteria” contributing to CRC may be different between enterotypes^23,26^. The *Prevotella* enterotype is dominated by fibre-using bacteria that ferment dietary fibre into short chain fatty acids (SCFAs)^14^. Subjects with the *Prevotella* enterotype have been reported to have a lower serum low-density lipoprotein level, which is associated with a lower cardiometabolic risk^30^. Metabolic syndrome is a risk factor for the incidence and recurrence of CRC and is a poor prognostic factor after radical resection^31,32^. In our Prevotella-enterotype cohort, enriched *Fusobacterium* and depleted *Coprococcus* levels were consistent with the results of a previous study that analysed stool and mucosa samples from CRC patients^15^. *Coprococcus* is a butyrate-producing anaerobe with immunomodulatory and anti-inflammatory properties^33^. *Coprococcus comes* is associated with a healthy gut and is particularly common in healthy Mongolians^34^. This finding may play a key role in the pathogenesis of CRC in this enterotype.

To date, no study has reported on microbiota changes during the transition sequence from advanced adenoma to carcinoma *in situ* to early CRC. For the first time, we found that *Oscillospira* levels were significantly reduced in stage 0 CRC, whereas *Haemophilus* was reduced in early-stage CRC. *Oscillospira* are under-studied anaerobes and butyrate-producing bacteria associated with leanness that have been found to be reduced in humans in the setting of inflammation^35,36^. The two genera may act as competitors in the healthy gut. The increasing richness of these organisms during the transition from pre-cancerous lesions to late-stage CRC may arise from the overgrowth of harmful bacteria as sequela of the two depleted taxa.

A bacterial driver-passenger model was previously proposed for CRC to explain individual variations between CRC patients and healthy subjects^37^. The gut microbiota of CRC patients carries more “driver” bacteria with pro-carcinogenic features that can interact with the intestinal microenvironment but are then outcompeted by “passenger” bacteria. In our study, the abundance of *Bacteroides* and *Citrobacter* in enterotype 1 and *Bacteroides*, *Eubacterium*, *Faecalibacterium*, *Ruminococcus*, *Bilophila*, and *Roseburia* in enterotype 3 decreased along the adenoma-carcinoma sequence, suggesting that these bacteria act as “driver” bacteria, a finding consistent with that of a previous report^37^. In addition, increases in *Fusobacterium* and *Clostridium* in enterotype 2 and enterotype 3 and *Pseudomonas*, *Aeromonas*, and *Porphyromonas* in enterotype 3 in the CRC group were consistent with “passenger” bacteria as sequela of CRC.

A strength of our study is that we systemically analysed different stages of colorectal neoplasms along the conventional adenoma-carcinoma sequence and across different enterotypes^11^. Our findings confirmed that faecal microbiota is a potentially favourable detection tool for CRC. Current screening tools use FIT, which detects human globin and is less influenced by diet or drugs^7^. However, the sensitivity of FIT studies varies from 65%-81% for CRC and is less than 30% for advanced neoplasms, which need detectable haemoglobin in the stool for increased accuracy^7^. The multi-target stool DNA test improves the cancer detection rate, with a sensitivity of 92.3% for CRC and 42.4% for pre-cancerous lesions^10^. The DNA test detects gene mutations presented in a shedding adenoma or tumour that improves its diagnostic accuracy in the setting of CRC. However, this test is still limited in the setting of non-cancerous neoplasms, and its accuracy may also be confounded by tumour size. Combined, the faecal metagenomic test and FIT might improve CRC detection sensitivity dramatically^22^. It is important to perform further validation tests, and the addition of an enterotype analysis should be considered. Furthermore, to increase the clinical value of such tests, it is necessary to develop affordable stool tests combined with a stool occult blood test. The ultimate goal is to provide a more predictive non-invasive screening tool, which may increase patient interest in receive screening tests and reduce clinical load and medical resource cost.

Our study was limited by the small number of subjects in enterotype 2. Second, this is an observational study, and the CAG classifiers need further validation and comparison with FIT. Third, a more cost-effective method is required for clinical translation.

In conclusion, we performed an enterotype-based analysis of CRC human faecal microbiota along the conventional adenoma-carcinoma sequence. We highlight that the dysbiotic features of CRC luminal gut microbiota are different across enterotypes, implying that our results may be confounded by lifestyle and long-term dietary habits. Although the interaction involving the process of carcinogenesis and CRC progression requires further study on the tissue microbiota, faecal microbiota could be a potential tool for the screening of CRC. To improve their predictive value, metagenomic biomarkers may not be composed of a single gene or taxon. A combination of CAG with known increased or decreased abundance should be evaluated. Future studies should include the validation of biomarkers in a different cohort and a comparison with current screening and diagnostic approaches.

## Methods

### Ethics approval and consent to participate

This study was reviewed and approved by the Ethics Committee of Chung Shan Medical University Hospital (CSMUH No: CS14047). All of the methods were performed in accordance with relevant guidelines and regulations, including any relevant details. Informed consents were obtained from all patients, as approved by the Institutional Review Board.

### Patients and sample collection

From 2014 to 2016, 283 participants underwent a screening or surveillance colonoscopy were enrolled at Chung Shan Medical University Hospital, Taichung, Taiwan. All fresh faecal samples were collected from the patients before colonoscopy using Sigma-Transwab (Medical Wire, Corsham, Wiltshire England) with Liquid Amies Transport Medium before their colon preparation procedure and were stored in their home refrigerators at −20 °C prior to transport to the laboratory, where the samples were stored in a freezer at −80 °C. Subjects who were under the age of forty, pregnant, used antibiotics or probiotics within two months of stool collection, had evidence of infection, had undergone a colectomy, received preoperative chemotherapy or radiotherapy, or were diagnosed with inflammatory bowel disease (e.g., Crohn’s disease or ulcerative colitis) or any malignancy were excluded from the study.

### Bowel preparation, colonoscopy, and pathology

All participants underwent a conventional bowel preparation that included polyethylene glycol electrolyte lavage powder (containing sodium chloride 21.36 mg, sodium bicarbonate 24.57 mg, potassium chloride 10.83 mg, sodium sulfate anhydrous 82.9 mg, and polyethylene glycol 4000 860.34 mg). Colonoscopies were performed primarily by 7 experienced endoscopists. Based on the colonoscopy findings and pathology reports, subjects were grouped into normal, small adenoma, advanced adenoma (i.e., size ≥1 cm, villous or tubulovillous features, or high grade dysplasia), carcinoma *in situ* (stage 0), early-stage carcinoma (stage I and II), and late-stage carcinoma (stage III and IV) groups^38^. Patients who did not receive a complete colonoscopy or had serrated polyps were also excluded from the study.

### DNA extraction

In this study, faeces were obtained from the participants. DNA was extracted directly from the stool samples using a QIAamp Fast DNA Stool Mini Kit (Qiagen, Hilden, Germany). For stool samples, a swab was vortexed vigorously and incubated at room temperature for 1 min. An aliquot of 200 μL of each sample was then transferred a microcentrifuge tube containing 950 μL InhibitEX Buffer and then vortexed until it was thoroughly homogenized. An enzyme solution (50 μL of 4 mg/mL lysozyme; 4 mM Tris·HCl, pH 8.0; 0.4 mM EDTA; 0.4% SDS) was added into the sample, which was then incubated at 37 °C for 30 min and 95 °C for 15 min. Particles were pelleted with a centrifuge, and 600 μL of supernatant was transferred into a new tube that contained 45 μL of proteinase K (20 mg/mL) and 600 μL of Buffer AL. After 10 minutes of incubation at 70 °C, 600 μL of ethanol was added to the lysate. Extractions were then performed with QIAamp spin columns according to the QIAamp Fast DNA Stool Mini Kit protocol. The extracted DNA from the stool was eluted with 50 μL Buffer AE. All samples were centrifuged at 18,000 × *g* for 1 min. Final concentrations were measured using a NanoPhotometer (Implen, Westlake Village, CA USA) and then stored at −20 °C for further analysis.

### Library construction and sequencing for the V3-V4 region of the 16S ribosomal RNA gene

The 16S rRNA gene, a molecular marker for identifying bacterial species, consists of nine hypervariable regions. Using 2-step PCR amplification, we can add adaptor sequences into the V3 and V4 hypervariable regions. This region, which provides ample information on the taxonomic classification of microbial communities from specimens associated with human microbiome studies, was used in the Human Microbiome Project^39^.

The 1st step of PCR is to amplify the V3 and V4 hypervariable regions. The amplicon primers are designed to contain (1) gene-specific sequences selected from work done by Klindworth *et al*.^40^; (2) a sequencing primer binding site that allows amplicons to be sequenced via dual-indexed sequencing with the MiSeq system (Illumina, San Diego, CA USA); and 3) a 0 to 7 bp “heterogeneity spacer” that increases the sequence diversity of the 16S rRNA gene libraries^41^. PCR amplification was performed using a 25 μL reaction volume that contained 12.5 μL of 2X KAPA HiFi HotStart ReadyMix (KAPA Biosystems, Wilmington, MA USA), 0.2 μM each of forward and reverse primer, and 100 ng of the DNA template. The reaction process was executed by raising the solution temperature to 95 °C for 3 min, then performing 25 cycles of 98 °C for 20 sec, 55 °C for 30 sec, and 72 °C for 30 sec, ending with the temperature held at 72 °C for 5 min. Amplicons were purified using the AMPure XP PCR Purification Kit (Beckman Coulter Life Sciences, Indianapolis, IN USA).

The second step of PCR is to add the index adaptors using a 10-cycle PCR programme. The PCR step adds the index 1 (i7), index 2 (i5), sequencing, and common adapters (P5 and P7) required for cluster generation and sequencing. PCR amplification was performed on a 25 μL reaction volume containing 12.5 μL of 2X KAPA HiFi HotStart ReadyMix (KAPA Biosystems, Wilmington, MA USA), 0.2 μM of each index adaptor (i5 and i7), and 2.5 μL of the first-PCR final product. The reaction process was executed by raising the solution temperature to 95 °C for 3 min, then performing 10 cycles of 98 °C for 20 sec, 55 °C for 30 sec, and 72 °C for 30 sec, ending with a 72 °C hold for 5 min. Amplicons were purified using the AMPure XP PCR Purification Kit (Beckman Coulter Life Sciences, Indianapolis, IN USA).

Amplified products were then checked with 2% agarose gel electrophoresis with Novel Juice (GeneDireX, Taiwan). Amplicons were purified using the AMPure XP PCR Purification Kit (Beckman Coulter Genomics, Danvers, MA, USA) and quantified using the Qubit dsDNA HS Assay Kit and a Qubit 2.0 Fluorimeter (Thermo Fisher Scientific, Waltham, MA USA), and qPCR with the Library Quantification Kit for Illumina (KAPA Biosystems, Wilmington, MA USA), all according to their corresponding manufacturer’s instructions.

The PhiX Control library (v3) (Illumina, San Diego, CA USA) was combined with the amplicon library (expected at 20%). The library was clustered to a density of approximately 800–1000 K/mm^2^. The libraries were processed for cluster generation and sequencing on 250PE MiSeq runs, and one library was sequenced using the standard Illumina sequencing primers, eliminating the need for an eight-index read. Sequencing data were available within approximately 40 h. Image analysis, base calling and data quality assessment were performed using the MiSeq instrument.

### 16S rRNA gene V3V4 region amplicon sequencing data quality control

Heterogeneity spacers^41^ and 5′ end primer sequence were identified and removed by in-house script. FASTX-Toolkit (http://hannonlab.cshl.edu/fastx_toolkit) was applied to control that the read quality in 70% or above of read region of each read is higher than Q20. We also applied fastq_quality_trimmer from FASTX-Toolkit to cut the bad quality 3′ tail of each read, and remain the read which length is higher than 100 nts. Finally, we matched read 1 (forward read) and read 2 (reverse reads) for next taxonomy assignment analysis stage.

### Taxonomy assignment and OTU table generation

Bowtie2 (2.2.8)^42^ was applied to align paired sequencing reads that passed quality control to a 16S rRNA gene sequence to reference, the SILVA database (release SILVA_SSU_Parc_115)^43,44^. We set the parameters were “–very-sensitive–end-to-end–no-mixed–no-discordant–dovetail -X 1000” to make the alignment results with higher specificity. We assigned the taxonomy when both paired reads are 97% or above similarity to the same taxonomy reference. After this taxonomy assignment step, an operational taxonomic unit (OTU) table was generated.

### Downstream analysis

#### Enterotyping based on the relative abundance of Bacteroides and Prevotella

To create a genus-level OTU table, OTUs with the same genus name were merged into one genus. We then calculated the relative abundance of each genus. We classified three enterotypes based on the following criteria: (i) Enterotype I, ***RA***_***B***_ ≥ 40% and ***RA***_***B***_ > ***RA***_***p***_; (ii) Enterotype II, ***RA***_***p***_ ≥ 30% and ***RA***_***p***_ ≥ ***RA***_***B***_; (iii) Enterotype III, Others. If the ***RA***_***B***_ in one sample was greater than or equal to 40% and the ***RA***_***B***_ was greater than the ***RA***_***P***_, the sample was classified as enterotype I. If the ***RA***_***P***_ was greater than or equal to 30% and the ***RA***_***P***_ was greater than or equal to the ***RA***_***B***_, the sample was classified as enterotype II. Otherwise, the sample was placed into the enterotype III group. ***RA***_***B***_ represents the relative abundance of *Bacteroides*, and ***RA***_***P***_ represents the relative abundance of *Prevotella*.

### Statistical analysis

The richness and Shannon index was used to calculate alpha-diversity. A two-sided Mann-Whitney rank test (python package SciPy 1.0.0) was used to compare the two groups. A correlation analysis using Spearman’s rank correlation coefficient was performed using the corrplot package in R (R Foundation for Statistical Computing, Vienna, Austria), and the co-abundancegroups (CAGs) were defined by the corrplot created heat plots (Figs S4A, S5A, S6A and S7A) and the hierarchical clustering in plots^15,45^. Tax4Fun^46^ was used to predict the function and metabolic capabilities of the microbial communities.

### Visualization

R software and the ggplot2 and reshape2 packages were used to create boxplots. A heatmap of our functional analysis was illustrated with MORPHEUS (https://software.broadinstitute.org/morpheus). The correlation network of specific genera was built using Cytoscape^47^.

## Supplementary information

Dataset 1

## Data Availability

Sequence data associated with this project have been deposited at the NCBI under study accession SRP131074 (https://www.ncbi.nlm.nih.gov/sra/SRP131074).
