# Supplementary material for: Enterotype-based Analysis of Gut Microbiota along the Conventional Adenoma-Carcinoma Colorectal Cancer Pathway
Source: Sci Rep. 2019 Jul 29;9:10923. doi: 10.1038/s41598-019-45588-z (PMC6662695; doi:10.1038/s41598-019-45588-z)
Supplement: Supplementary file 1 — Dataset 1 [file 41598_2019_45588_MOESM1_ESM.pdf]

Enterotype-based Analysis of Gut Microbiota along the Conventional Adenoma-Carcinoma Colorectal Cancer Pathway

Tzu-Wei Yang, M.D.[1,4,5†], Wei-Hsiang Lee, Ph. D.[5,6,7†], Siang-Jyun Tu[7], Wei-Chih Huang, Ph. D.[5,6,7], Hui-Mei Chen[7], Ting-Hsuan Sun[5], Ming-Chang Tsai, M.D.[1,3], Chi-Chih Wang, M.D.[1,3], Hsuan-Yi Chen, M.D.[1,4], Chi-Chou Huang, M.D.[2,4], Bei-Hao Shiu, M.D.[2,3], Tzu-Ling Yang[5], Hsin-Tzu Huang[5], Yu-Pao Chou[7], Chih-Hung Chou, Ph. D.[5,7], Ya-Rong Huang[7], Yi-Run Sun[7], Chao Liang[7], Feng-Mao Lin, Ph. D.[7], Shinn-Ying Ho, Ph. D.[5,7], Wen-Liang Chen, Ph. D.[5], Shun-Fa Yang, Ph. D.[3,6], Kwo-Chang Ueng, M.D., Ph. D.[4,6], Hsien-Da Huang, Ph. D.[5,7*], Chien-Ning Huang, M.D., Ph. D.[8*], Yuh-Jyh Jong, M.D., D. M. Sci.[5,9,10,11*], Chun-Che Lin, M.D., Ph. D.[1,4*]

[1]Division of Gastroenterology and Hepatology, Department of Internal Medicine, Chung Shan Medical University Hospital, Taichung 402, Taiwan

[2]Division of Colon and Rectum, Department of Surgery, Chung Shan Medical University Hospital, Taichung, Taiwan.

[3]Institute of Medicine, Chung Shan Medical University, Taichung 402, Taiwan

[4]School of Medicine, Chung Shan Medical University, Taichung 402, Taiwan

[5]Institute and Department of Biological Science and Technology, College of Biological Science and Technology, National Chiao Tung University, Hsinchu, Taiwan

[6]Department of Medical Research, Chung Shan Medical University Hospital,

Taichung 402, Taiwan

[7]Institute of Bioinformatics and Systems Biology, College of Biological Science and Technology, National Chiao Tung University, Hsinchu 300, Taiwan

[8]Division of Endocrinology and Metabolism, Department of Internal Medicine, Chung Shan Medical University Hospital, Taichung 402, Taiwan

[9]Graduate Institute of Clinical Medicine, College of Medicine, Kaohsiung Medical University, Kaohsiung 807, Taiwan

[10]Departments of Pediatrics and Laboratory Medicine, Kaohsiung Medical University Hospital, Kaohsiung Medical University, Kaohsiung 807, Taiwan

[11]Institute of Molecular Medicine and Bioengineering, College of Biological Science and Technology, National Chiao Tung University, Hsinchu, Taiwan

*Correspondence: bryan@mail.nctu.edu.tw; cshy049@gmail.com; yjjongnctu@gmail.com; forest65@csmu.edu.tw

†These authors contributed equally to this work.

Supplementary Tables

Table S1: Phylum-level microbial composition *Bcateroidetes, Proteobacteria,* and *Firmicutes* are the most dominant phyla in stool samples. These 3 phyla covered more than 95% of our sequenced reads. Total 10 phyla exist in all groups, except the phylum, *Gemmatimonadetes*, only found in cancer group. The 10 phyla share the same relative abundance order in three groups with minor variations of percentages.

Phylum	All	Normal	Adenoma	Cancer
Bacteroidetes	48.998%	48.121%	53.856%	45.017%
Proteobacteria	32.947%	34.744%	28.328%	35.769%
Firmicutes	13.118%	13.103%	14.666%	11.584%
Verrucomicrobia	2.540%	2.022%	1.734%	3.862%
Fusobacteria	1.334%	1.277%	0.679%	2.046%
Actinobacteria	0.809%	0.591%	0.650%	1.184%
Synergistetes	0.202%	0.083%	0.047%	0.476%
Euryarchaeota	0.041%	0.058%	0.036%	0.030%
Deferribacteres	0.011%	0.002%	0.002%	0.029%
Tenericutes	3.340E-06	6.352E-07	3.929E-07	0.001%
Gemmatimonadetes	0.001%	0	0	0.002%

Table S2. Major common bacteria Top 20 genera in all and each groups based on relative abundance. All groups share 16 common genera with different order. All these 25 genera occupied more than 90% of the relative abundance in each group.

Genus	All	Normal	Adenoma	Cancer	All	Normal	Adenoma	Cancer
Bacteroides	36.52%	34.68%	39.17%	30.98%	1	1	1	1
Escherichia	16.03%	18.96%	13.98%	18.85%	2	2	2	2
Prevotella	9.84%	9.72%	10.12%	9.25%	3	3	3	3
Klebsiella	4.63%	4.06%	5.82%	3.19%	4	5	4	6
Shigella	3.86%	4.58%	3.38%	4.52%	5	4	5	4
Alistipes	2.53%	2.20%	2.53%	2.42%	6	6	7	7
Faecalibacterium	2.42%	2.10%	3.05%	1.83%	7	7	6	9
Akkermansia	2.27%	2.02%	1.73%	3.86%	8	9	11	5
Ruminococcus	1.92%	1.76%	2.27%	1.55%	9	12	8	11
Clostridium	1.84%	2.02%	1.75%	1.83%	10	10	10	10
Enterobacter	1.64%	1.87%	1.27%	1.33%	11	11	14	15
Eubacterium	1.55%	1.27%	1.92%	1.05%	12	15	9	18
Veillonella	1.51%	2.09%	1.52%	1.39%	13	8	13	13
Parabacteroides	1.41%	1.20%	1.71%	1.16%	14	16	12	17
Roseburia	1.14%	1.17%	1.09%	0.96%	15	17	16	19
Fusobacterium	1.11%	1.28%	0.68%	2.05%	16	14	18	8
Citrobacter	1.05%	1.40%	1.25%	0.35%	17	13	15	

Bilophila	0.68%	0.85%	0.62%	0.55%	18	18	20	
Pseudomonas	0.60%	0.72%	0.07%	1.18%	19	19		16
Blautia	0.48%	0.71%	0.44%	0.33%	20	20		
Streptococcus	0.48%	0.38%	0.65%	0.47%			19	
Oscillospira	0.42%	0.24%	0.69%	0.25%			17	
Aeromonas	0.38%	0.06%	0.16%	1.43%				12
Morganella	0.34%	0.06%	0.03%	1.34%				14
Odoribacter	0.31%	0.19%	0.22%	0.65%				20

Table S3. Summary of case numbers in 3 enterotypes

	Total	Nomral	Adenoma	Colorectal cancer
Enterotype 1 (N, %) *Bacteroides* - dominated	123	42 (34.15)	57 (46.34)	24 (19.51)
Enterotype 2 (N, %) *Prevotella* - dominated	36	13 (36.11)	16 (44.44)	7 (19.44)
Enterotype 3 (N, %) *Escherichia* - dominated	124	49 (39.52)	44 (35.48)	31 (25)

Table S4A. The statistically significant genera in overall samples: Mann-Whitney U test Single genus was selected by applying Mann-Whitney U test on relative abundance, and each genus presents in more than 50% of samples in cancer group. Most of the significances are between cancer group and other 2 groups. ($p < 0.01$)

(AP_A: Appearance Percentage of Adenoma, AP_C: Appearance Percentage of Cancer, AP_N: Appearance Percentage of Normal, AP_T: Appearance Percentage of Total)

(MR_A: Mean Reads of Adenoma, MR_C: Mean Reads of Cancer, MR_N: Mean Reads of Normal, MR_T: Mean Reads of Total)

(p_AC: p-value of Utest of Adenoma & Cancer, p_AN: p-value of Utest of Adenoma & Normal, p_CN: p-value of Utest of Cancer & Normal)

(FDR_AC: adjust p-value of Utest of Adenoma & Cancer, FDR_AN: adjust p-value of Utest of Adenoma & Normal, FDR_CN: adjust p-value of Utest of Cancer & Normal)

Genus	AP_A	AP_C	AP_N	AP_T	MR_A	MR_C	MR_N	MR_T	p_AC	FDR_AC	p_AN	FDR_AN	p_CN	FDR_CN
Fusobacterium	93.16%	100.00%	87.50%	92.58%	295.55	733.95	579.68	496.01	6.75E-04	2.62E-03	1.16E-01	4.90E-01	3.48E-05	3.73E-04
Eubacterium	100.00%	100.00%	100.00%	100.00%	835.32	375.23	577.97	639.95	7.69E-04	2.91E-03	3.82E-01	6.02E-01	9.04E-03	2.94E-02
Roseburia	100.00%	98.39%	99.04%	99.29%	473.84	345.6	529.88	466.34	2.08E-03	7.15E-03	6.10E-01	8.01E-01	6.45E-03	2.36E-02
Bacteroides	100.00%	100.00%	100.00%	100.00%	17041.4	11113.27	15749.6	15267.94	8.59E-05	4.76E-04	1.27E-01	4.90E-01	9.22E-03	2.94E-02
Dorea	94.87%	87.10%	98.08%	94.35%	86.74	57.52	93.29	82.75	4.00E-03	1.26E-02	5.01E-01	7.10E-01	2.74E-03	1.11E-02
Enterococcus	41.88%	62.90%	50.96%	49.82%	3.47	86.1	38.5	34.45	2.93E-03	9.52E-03	8.50E-02	4.90E-01	1.54E-01	2.77E-01
Parabacteroides	100.00%	100.00%	100.00%	100.00%	742.35	415.21	545.13	598.2	1.12E-03	4.13E-03	3.78E-01	6.01E-01	1.44E-02	4.26E-02
Coprococcus	77.78%	72.58%	75.96%	75.97%	72.53	31.52	58.62	58.43	8.90E-03	2.53E-02	1.65E-01	5.56E-01	1.39E-01	2.53E-01
Ruminococcus	100.00%	100.00%	100.00%	100.00%	986.32	555.27	798.16	822.74	2.81E-03	9.34E-03	2.16E-01	5.62E-01	4.46E-02	1.03E-01
Faecalibacterium	100.00%	100.00%	100.00%	100.00%	1328.11	656.24	954.17	1043.5	2.87E-03	9.43E-03	3.01E-01	5.69E-01	2.44E-02	6.23E-02
Morganella	30.77%	50.00%	38.46%	37.81%	14.81	479.89	25.68	120.7	8.85E-03	2.53E-02	2.53E-01	5.62E-01	1.01E-01	1.94E-01
Cronobacter	100.00%	98.39%	98.08%	98.94%	98.31	85.23	157.84	117.32	1.99E-01	2.90E-01	1.14E-03	1.14E-01	1.51E-04	9.85E-04
Pseudomonas	78.63%	85.48%	92.31%	85.16%	28.68	423.02	325.22	224.05	5.13E-02	1.04E-01	9.73E-03	2.89E-01	9.96E-01	9.96E-01
Bilophila	96.58%	93.55%	95.19%	95.41%	270.86	197.82	387.91	297.88	1.56E-02	4.03E-02	1.66E-01	5.56E-01	6.25E-04	3.29E-03
Aeromonas	74.36%	79.03%	72.12%	74.56%	68.49	514.79	29.2	151.83	2.94E-01	3.96E-01	1.58E-02	3.23E-01	2.18E-03	9.21E-03
Citrobacter	97.44%	95.16%	95.19%	96.11%	545.99	126.87	634.11	486.55	1.67E-02	4.03E-02	3.46E-02	3.23E-01	5.44E-05	5.10E-04

Table S4B. The statistically significant genera in enterotypes I: Mann-Whitney U test Single genus was selected by applying Mann-Whitney U test on relative abundance, and each genus presents in more than 50% of samples in cancer group. The significances of *Citrobacter* are between cancer group and other 2 groups, while *Bacteroides* is significant among cancer and adenoma. ($p < 0.01$)
(AP_A: Appearance Percentage of Adenoma, AP_C: Appearance Percentage of Cancer, AP_N: Appearance Percentage of Normal, AP_T: Appearance Percentage of Total)
(MR_A: Mean Reads of Adenoma, MR_C: Mean Reads of Cancer, MR_N: Mean Reads of Normal, MR_T: Mean Reads of Total)
(p_AC: p-value of Utest of Adenoma & Cancer, p_AN: p-value of Utest of Adenoma & Normal, p_CN: p-value of Utest of Cancer & Normal)
(FDR_AC: adjust p-value of Utest of Adenoma & Cancer, FDR_AN: adjust p-value of Utest of Adenoma & Normal, FDR_CN: adjust p-value of Utest of Cancer & Normal)

Genus	AP_A	AP_C	AP_N	AP_T	MR_A	MR_C	MR_N	MR_T	p_AC	FDR_AC	p_AN	FDR_AN	p_CN	FDR_CN
Citrobacter	98.25%	100.00%	95.24%	97.56%	170.44	106.25	340.14	215.86	9.34E-03	8.95E-02	8.92E-02	7.93E-01	2.79E-03	2.87E-01
Bacteroides	100.00%	100.00%	100.00%	100.00%	24114.7	19804	24986	23571.1	9.57E-03	8.95E-02	9.29E-01	9.88E-01	2.43E-02	2.93E-01

Table S4C. The statistically significant genera in enterotypes II: Mann-Whitney U test Single genus was selected by applying Mann-Whitney U test on relative abundance, and each genus presents in more than 50% of samples in cancer group. The significance of *Fusobacterium* is between cancer and normal group, while *Coprococcus* is between cancer and adenoma, and *Clostridium* is among normal and adenoma groups. ($p < 0.01$)

(AP_A: Appearance Percentage of Adenoma, AP_C: Appearance Percentage of Cancer, AP_N: Appearance Percentage of Normal, AP_T: Appearance Percentage of Total)

(MR_A: Mean Reads of Adenoma, MR_C: Mean Reads of Cancer, MR_N: Mean Reads of Normal, MR_T: Mean Reads of Total)

(p_AC: p-value of Utest of Adenoma & Cancer, p_AN: p-value of Utest of Adenoma & Normal, p_CN: p-value of Utest of Cancer & Normal)

(FDR_AC: adjust p-value of Utest of Adenoma & Cancer, FDR_AN: adjust p-value of Utest of Adenoma & Normal, FDR_CN: adjust p-value of Utest of Cancer & Normal)

Genus	AP_A	AP_C	AP_N	AP_T	MR_A	MR_C	MR_N	MR_T	p_AC	FDR_AC	p_AN	FDR_AN	p_CN	FDR_CN
Coprococcus	100.00%	85.71%	76.92%	88.89%	163.94	8.43	47.08	91.5	6.75E-03	3.61E-01	4.12E-02	6.84E-01	3.19E-01	6.93E-01
Clostridium	100.00%	100.00%	100.00%	100.00%	720.38	560.43	248.46	518.86	8.15E-01	9.21E-01	9.07E-03	6.84E-01	9.61E-02	6.75E-01
Fusobacterium	93.75%	100.00%	76.92%	88.89%	120.94	441.43	4.46	141.19	1.93E-02	4.73E-01	1.08E-02	6.84E-01	4.63E-04	6.16E-02

Table S4D. The statistically significant genera in enterotypes III: Mann-Whitney U test Single genus was selected by applying Mann-Whitney U test on relative abundance, and each genus presents in more than 50% of samples in cancer group. Most of the significances are between cancer group and other 2 groups. ($p < 0.01$)

(AP_A: Appearance Percentage of Adenoma, AP_C: Appearance Percentage of Cancer, AP_N: Appearance Percentage of Normal, AP_T: Appearance Percentage of Total)

(MR_A: Mean Reads of Adenoma, MR_C: Mean Reads of Cancer, MR_N: Mean Reads of Normal, MR_T: Mean Reads of Total)

(p_AC: p-value of Utest of Adenoma & Cancer, p_AN: p-value of Utest of Adenoma & Normal, p_CN: p-value of Utest of Cancer & Normal)

(FDR_AC: adjust p-value of Utest of Adenoma & Cancer, FDR_AN: adjust p-value of Utest of Adenoma & Normal, FDR_CN: adjust p-value of Utest of Cancer & Normal)

Genus	AP_A	AP_C	AP_N	AP_T	MR_A	MR_C	MR_N	MR_T	p_AC	FDR_AC	p_AN	FDR_AN	p_CN	FDR_CN
Acinetobacter	54.55%	74.19%	45.83%	56.10%	115.8	67.68	93.04	94.79	7.99E-02	1.66E-01	1.81E-01	6.05E-01	1.96E-03	9.03E-03
Aeromonas	84.09%	80.65%	72.92%	78.86%	9.66	979.58	46.65	268.54	4.39E-01	5.20E-01	2.33E-02	5.59E-01	9.98E-03	3.35E-02
Arthrobacter	6.82%	51.61%	14.58%	21.14%	0.16	67.29	1.62	17.65	4.72E-06	1.39E-04	2.13E-01	6.05E-01	1.46E-04	1.72E-03
Bacillus	75.00%	83.87%	81.25%	79.67%	6.45	111.71	9.85	34.31	8.30E-03	2.53E-02	6.68E-02	6.05E-01	1.18E-01	2.32E-01
Bacteroides	100.00%	100.00%	100.00%	100.00%	10840.1	5410.97	9636.83	9002.22	3.20E-05	3.71E-04	1.51E-01	6.05E-01	2.16E-03	9.80E-03
Bilophila	93.18%	93.55%	95.83%	94.31%	270.93	165.68	497.81	332.94	2.70E-02	6.87E-02	1.43E-01	6.05E-01	6.89E-04	3.99E-03
Blautia	100.00%	100.00%	100.00%	100.00%	161.75	73.71	476.69	262.46	1.71E-02	4.59E-02	5.32E-01	7.98E-01	2.91E-03	1.26E-02
Brevibacillus	9.09%	58.06%	18.75%	25.20%	0.09	94.35	1.35	24.34	1.15E-06	6.67E-05	1.57E-01	6.05E-01	4.35E-05	1.07E-03
Brevundimonas	6.82%	54.84%	8.33%	19.51%	0.07	29.9	7.23	10.38	1.80E-06	8.70E-05	7.53E-01	9.57E-01	6.29E-06	6.19E-04
Delftia	31.82%	61.29%	33.33%	39.84%	0.55	34.48	1.38	9.42	4.02E-04	2.24E-03	7.07E-01	9.35E-01	8.29E-04	4.61E-03
Dorea	95.45%	80.65%	97.92%	92.68%	101.82	43.23	82.6	79.55	4.22E-04	2.24E-03	1.98E-01	6.05E-01	1.95E-02	5.43E-02
Enterococcus	47.73%	74.19%	50.00%	55.28%	4.95	159.84	78.25	72.59	3.25E-03	1.18E-02	4.88E-01	7.69E-01	1.78E-02	5.15E-02

Genus	AP_A	AP_C	AP_N	AP_T	MR_A	MR_C	MR_N	MR_T	p_AC	FDR_AC	p_AN	FDR_AN	p_CN	FDR_CN
Eubacterium	100.00%	100.00%	100.00%	100.00%	780.84	258.16	560.75	563.22	2.68E-03	1.02E-02	7.10E-01	9.35E-01	5.33E-03	1.89E-02
Faecalibacterium	100.00%	100.00%	100.00%	100.00%	1309.48	616.29	737.92	911.72	4.50E-03	1.59E-02	2.23E-01	6.05E-01	3.29E-02	8.16E-02
Fusobacterium	90.91%	100.00%	91.67%	93.50%	311.98	852.16	553.94	542.54	6.78E-03	2.09E-02	9.03E-01	9.75E-01	1.25E-02	3.72E-02
Ochrobactrum	2.27%	54.84%	16.67%	21.14%	0.02	59.13	1.08	15.33	1.58E-07	3.75E-05	1.99E-02	5.37E-01	7.78E-05	1.18E-03
Parabacteroides	100.00%	100.00%	100.00%	100.00%	953.36	301.26	481.52	604.88	4.34E-04	2.25E-03	2.41E-01	6.05E-01	8.15E-03	2.83E-02
Porphyromonas	22.73%	54.84%	33.33%	34.96%	4.39	271.74	2.31	70.96	9.62E-04	4.50E-03	2.22E-01	6.05E-01	1.11E-02	3.52E-02
Pseudomonas	77.27%	83.87%	95.83%	86.18%	61.23	644.52	638.15	433.37	1.26E-02	3.69E-02	1.42E-03	1.53E-01	6.88E-01	7.78E-01
Roseburia	100.00%	96.77%	97.92%	98.37%	384.86	159.16	527.54	383.66	4.00E-03	1.43E-02	5.04E-01	7.89E-01	1.00E-02	3.35E-02
Ruminococcus	100.00%	100.00%	100.00%	100.00%	1124.32	424.06	648.75	762.24	3.25E-03	1.18E-02	1.33E-01	6.05E-01	3.29E-02	8.16E-02
Stenotrophomonas	11.36%	54.84%	25.00%	27.64%	0.48	106.58	13.6	32.34	1.33E-05	2.76E-04	9.05E-02	6.05E-01	1.15E-03	6.17E-03

Table S5. NC correlated CAGs The co-abundance groups (CAG) between the normals and CRCs clustered by Pearson's correlation analysis. Cluster 2 contained ten genera (red marked) which were clustered in every group with high correlation coefficient.

Cluster 1	Cluster 2	Cluster 3	Cluster 4	Cluster 5	Cluster 6	Cluster 7
Acinetobacter	*Brenneria*	*Actinomyces*	*Akkermansia*	*Collinsella*	*Lachnospira*	*Anaerostipes*
Aeromonas	*Cronobacter*	*Bifidobacterium*	*Alistipes*	*Coprococcus*	*Lactobacillus*	*Bacteroides*
Bacillus	*Erwinia*	*Eggerthella*	*Anaerotruncus*	*Coriobacterium*	*Prevotella*	*Bilophila*
Citrobacter	*Escherichia*	*Enterococcus*	*Desulfovibrio*	*Dorea*	*Roseburia*	*Blautia*
Enterobacter	*Nitrobacter*	*Fusobacterium*	*Lachnobacterium*	*Eubacterium*		*Clostridium*
Klebsiella	*Paracoccus*	*Granulicatella*	*Odoribacter*	*Faecalibacterium*		
Leclercia	*Pectobacterium*	*Haemophilus*	*Oscillospira*	*Ruminococcus*		
Pantoea	*Photorhabdus*	*Peptostreptococcus*	*Oxalobacter*	*Subdoligranulum*		
Pseudomonas	*Shigella*	*Streptococcus*	*Paludibacter*			
Raoultella	*Sporosarcina*	*Veillonella*	*Parabacteroides*			
Salmonella			*Synergistes*			
Serratia						
Xenorhabdus						

Table S6. Significances between NC correlated CAGs Clusters were selected by applying Mann-Whitney U test on summation of relative abundance. The significances of all samples are between adenomatous polyps and CRCs in Cluster 3 and Cluster 5. The significances of enterotype 3 samples are between adenomatous polyps and CRCs in Cluster 5 and Cluster 7. ($p < 0.01$)

(AP_A: Appearance Percentage of Adenoma, AP_C: Appearance Percentage of Cancer, AP_N: Appearance Percentage of Normal, AP_T: Appearance Percentage of Total)

(MR_A: Mean Reads of Adenoma, MR_C: Mean Reads of Cancer, MR_N: Mean Reads of Normal, MR_T: Mean Reads of Total)

(p_AC: p-value of Utest of Adenoma & Cancer, p_AN: p-value of Utest of Adenoma & Normal, p_CN: p-value of Utest of Cancer & Normal)

**All**	**AP_A**	**AP_C**	**AP_N**	**AP_T**	**MR_A**	**MR_C**	**MR_N**	**MR_T**	**p_AC**	**p_AN**	**p_CN**
Cluster3	100.00%	100.00%	100.00%	100.00%	487.44	673.61	554.29	552.79	6.72E-03	8.74E-01	1.97E-02
Cluster5	100.00%	100.00%	100.00%	100.00%	1112	760.73	901.85	957.81	7.10E-03	1.60E-01	1.22E-01
**E3**	**AP_A**	**AP_C**	**AP_N**	**AP_T**	**MR_A**	**MR_C**	**MR_N**	**MR_T**	**p_AC**	**p_AN**	**p_CN**
Cluster7	100.00%	100.00%	100.00%	100.00%	3980.59	2617	3506.62	3451.96	6.59E-03	1.31E-01	5.08E-02
Cluster5	100.00%	100.00%	100.00%	100.00%	1113.66	683.45	773.25	872.39	5.42E-03	2.23E-01	5.14E-02

Table S7. NAC correlated CAGs The co-abundance groups (CAG) between the normals, adenomatous polyps and CRCs clustered by Pearson's correlation analysis. Cluster 2 contained ten genera (red marked) which were clustered in every group with high correlation coefficient.

Cluster1	Cluster2	Cluster3	Cluster4	Cluster5	Cluster6	Cluster7
Acinetobacter	*Brenneria*	*Actinomyces*	*Desulfovibrio*	*Akkermansia*	*Prevotella*	*Anaerostipes*
Aeromonas	*Cronobacter*	*Enterococcus*	*Oscillospira*	*Alistipes*	*Lachnospira*	*Bifidobacterium*
Bacillus	*Erwinia*	*Granulicatella*	*Oxalobacter*	*Anaerotruncus*	*Lactobacillus*	*Bacteroides*
Citrobacter	*Escherichia*	*Haemophilus*	*Paludibacter*	*Odoribacter*	*Roseburia*	*Bilophila*
Enterobacter	*Nitrobacter*	*Peptostreptococcus*	*Synergistes*	*Parabacteroides*	*Ruminococcus*	*Blautia*
Klebsiella	*Paracoccus*	*Streptococcus*	*Collinsella*		*Dorea*	*Clostridium*
Leclercia	*Pectobacterium*	*Veillonella*	*Coriobacterium*		*Eubacterium*	*Eggerthella*
Pantoea	*Photorhabdus*		*Lachnobacterium*		*Faecalibacterium*	*Fusobacterium*
Pseudomonas	*Shigella*		*Coprococcus*			
Raoultella	*Sporosarcina*		*Subdoligranulum*			
Salmonella						
Serratia						
Xenorhabdus						

Table S8. Significances between NAC correlated CAGs Clusters were selected by applying Mann-Whitney U test on summation of relative abundance. The significances of enterotype 3 samples are between adenomatous polyps and CRCs in Cluster 6. ($p < 0.01$)
(AP_A: Appearance Percentage of Adenoma, AP_C: Appearance Percentage of Cancer, AP_N: Appearance Percentage of Normal, AP_T: Appearance Percentage of Total)
(MR_A: Mean Reads of Adenoma, MR_C: Mean Reads of Cancer, MR_N: Mean Reads of Normal, MR_T: Mean Reads of Total)
(p_AC: p-value of Utest of Adenoma & Cancer, p_AN: p-value of Utest of Adenoma & Normal, p_CN: p-value of Utest of Cancer & Normal)

E3	AP_A	AP_C	AP_N	AP_T	MR_A	MR_C	MR_N	MR_T	p_AC	p_AN	p_CN
Cluster6	100.00%	100.00%	100.00%	100.00%	2229.2	1298.52	2021.62	1913.63	3.25E-03	6.62E-01	6.71E-03

Table S9. NA correlated CAGs The co-abundance groups (CAG) between the normals and adenomatous polyps clustered by Pearson's correlation analysis. Cluster 2 contained ten genera (red marked) which were clustered in every group with high correlation coefficient.

Cluster1	Cluster2	Cluster3	Cluster4	Cluster5	Cluster6	Cluster7
Acinetobacter	*Brenneria*	*Actinomyces*	*Akkermansia*	*Lachnobacterium*	*Anaerostipes*	
Aeromonas	*Cronobacter*	*Enterococcus*	*Alistipes*	*Dorea*	*Bacteroides*	
Bacillus	*Erwinia*	*Granulicatella*	*Anaerotruncus*	*Coprococcus*	*Bifidobacterium*	
Citrobacter	*Escherichia*	*Haemophilus*	*Desulfovibrio*	*Eubacterium*	*Bilophila*	
Enterobacter	*Nitrobacter*	*Peptostreptococcus*	*Odoribacter*	*Faecalibacterium*	*Blautia*	
Klebsiella	*Paracoccus*	*Streptococcus*	*Oscillospira*	*Lachnospira*	*Clostridium*	
Leclercia	*Pectobacterium*	*Veillonella*	*Oxalobacter*	*Prevotella*	*Eggerthella*	
Pantoea	*Photorhabdus*	*Lactobacillus*	*Paludibacter*	*Roseburia*	*Fusobacterium*	
Pseudomonas	*Shigella*		*Synergistes*	*Subdoligranulum*		
Raoultella	*Sporosarcina*		*Collinsella*			
Salmonella			*Coriobacterium*			
Serratia			*Parabacteroides*			
Xenorhabdus			*Ruminococcus*			

Table S10. Significances between NA correlated CAGs Clusters were selected by applying Mann-Whitney U test on summation of relative abundance. The significances of enterotype 3 samples are between adenomatous polyps and CRCs, and Normals and CRCs, in Cluster 5. ($p < 0.01$)
(AP_A: Appearance Percentage of Adenoma, AP_C: Appearance Percentage of Cancer, AP_N: Appearance Percentage of Normal, AP_T: Appearance Percentage of Total)
(MR_A: Mean Reads of Adenoma, MR_C: Mean Reads of Cancer, MR_N: Mean Reads of Normal, MR_T: Mean Reads of Total)
(p_AC: p-value of Utest of Adenoma & Cancer, p_AN: p-value of Utest of Adenoma & Normal, p_CN: p-value of Utest of Cancer & Normal)

E3	AP_A	AP_C	AP_N	AP_T	MR_A	MR_C	MR_N	MR_T	p_AC	p_AN	p_CN
Cluster5	100.00%	100.00%	100.00%	100.00%	1937.39	1166.45	1847.81	1708.13	3.49E-03	8.02E-01	5.85E-03

Table S11. AC correlated CAGs The co-abundance groups (CAG) between the adenomatous polyps and CRCs clustered by Pearson's correlation analysis. Cluster 2 contained ten genera (red marked) which were clustered in every group with high correlation coefficient.

Cluster1	Cluster2	Cluster3	Cluster4	Cluster5	Cluster6	Cluster7
Acinetobacter	*Brenneria*	*Klebsiella*	*Akkermansia*	*Coprococcus*	*Collinsella*	*Bilophila*
Bacillus	*Cronobacter*	*Xenorhabdus*	*Alistipes*	*Dorea*	*Coriobacterium*	*Bacteroides*
Citrobacter	*Erwinia*	*Actinomyces*	*Desulfovibrio*	*Eubacterium*	*Prevotella*	*Blautia*
Leclercia	*Escherichia*	*Enterococcus*	*Odoribacter*	*Faecalibacterium*	*Anaerostipes*	*Clostridium*
Pseudomonas	*Nitrobacter*	*Granulicatella*	*Oscillospira*	*Lachnobacterium*	*Bifidobacterium*	*Eggerthella*
Raoultella	*Paracoccus*	*Streptococcus*	*Oxalobacter*	*Lachnospira*	*Haemophilus*	*Fusobacterium*
Serratia	*Pectobacterium*		*Paludibacter*	*Lactobacillus*	*Peptostreptococcus*	
Aeromonas	*Photorhabdus*		*Parabacteroides*	*Roseburia*	*Veillonella*	
Enterobacter	*Shigella*		*Synergistes*	*Ruminococcus*		
Pantoea	*Sporosarcina*		*Anaerotruncus*	*Subdoligranulum*		
Salmonella						

Table S12. Significances between AC correlated CAGs Clusters were selected by applying Mann-Whitney U test on summation of relative abundance. The significances of enterotype 3 samples are between adenomatous polyps and CRCs in Cluster 5. ($p < 0.01$)
(AP_A: Appearance Percentage of Adenoma, AP_C: Appearance Percentage of Cancer, AP_N: Appearance Percentage of Normal, AP_T: Appearance Percentage of Total)
(MR_A: Mean Reads of Adenoma, MR_C: Mean Reads of Cancer, MR_N: Mean Reads of Normal, MR_T: Mean Reads of Total)
(p_AC: p-value of Utest of Adenoma & Cancer, p_AN: p-value of Utest of Adenoma & Normal, p_CN: p-value of Utest of Cancer & Normal)

E3	AP_A	AP_C	AP_N	AP_T	MR_A	MR_C	MR_N	MR_T	p_AC	p_AN	p_CN
Cluster5	100.00%	100.00%	100.00%	100.00%	1248.23	726.55	952.33	1001.28	3.61E-03	4.21E-01	1.39E-02

Table S13. Most positively correlated genera (Cluster 2) Ten genera which were clustered in every group with high correlation coefficient. This table showed the correlated coefficient of these genera in cancer group.

Cancer	*Erwinia*	*Sporosarcina*	*Cronobacter*	*Paracoccus*	*Nitrobacter*	*Brenneria*	*Photorhabdus*	*Pectobacterium*	*Escherichia*	*Shigella*
Erwinia	1	0.637	0.623	0.619	0.622	0.655	0.663	0.682	0.671	0.680
Sporosarcina	0.637	1	0.664	0.736	0.706	0.750	0.742	0.761	0.764	0.763
Cronobacter	0.623	0.664	1	0.742	0.797	0.837	0.823	0.843	0.874	0.878
Paracoccus	0.619	0.736	0.742	1	0.842	0.873	0.876	0.876	0.877	0.867
Nitrobacter	0.622	0.706	0.797	0.842	1	0.915	0.921	0.924	0.921	0.914
Brenneria	0.655	0.750	0.837	0.873	0.915	1	0.947	0.948	0.961	0.954
Photorhabdus	0.663	0.742	0.823	0.876	0.921	0.947	1	0.954	0.959	0.951
Pectobacterium	0.682	0.761	0.843	0.876	0.924	0.948	0.954	1	0.971	0.966
Escherichia	0.671	0.764	0.874	0.877	0.921	0.961	0.959	0.971	1	0.997
Shigella	0.680	0.763	0.878	0.867	0.914	0.954	0.951	0.966	0.997	1

Supplementary Figures:

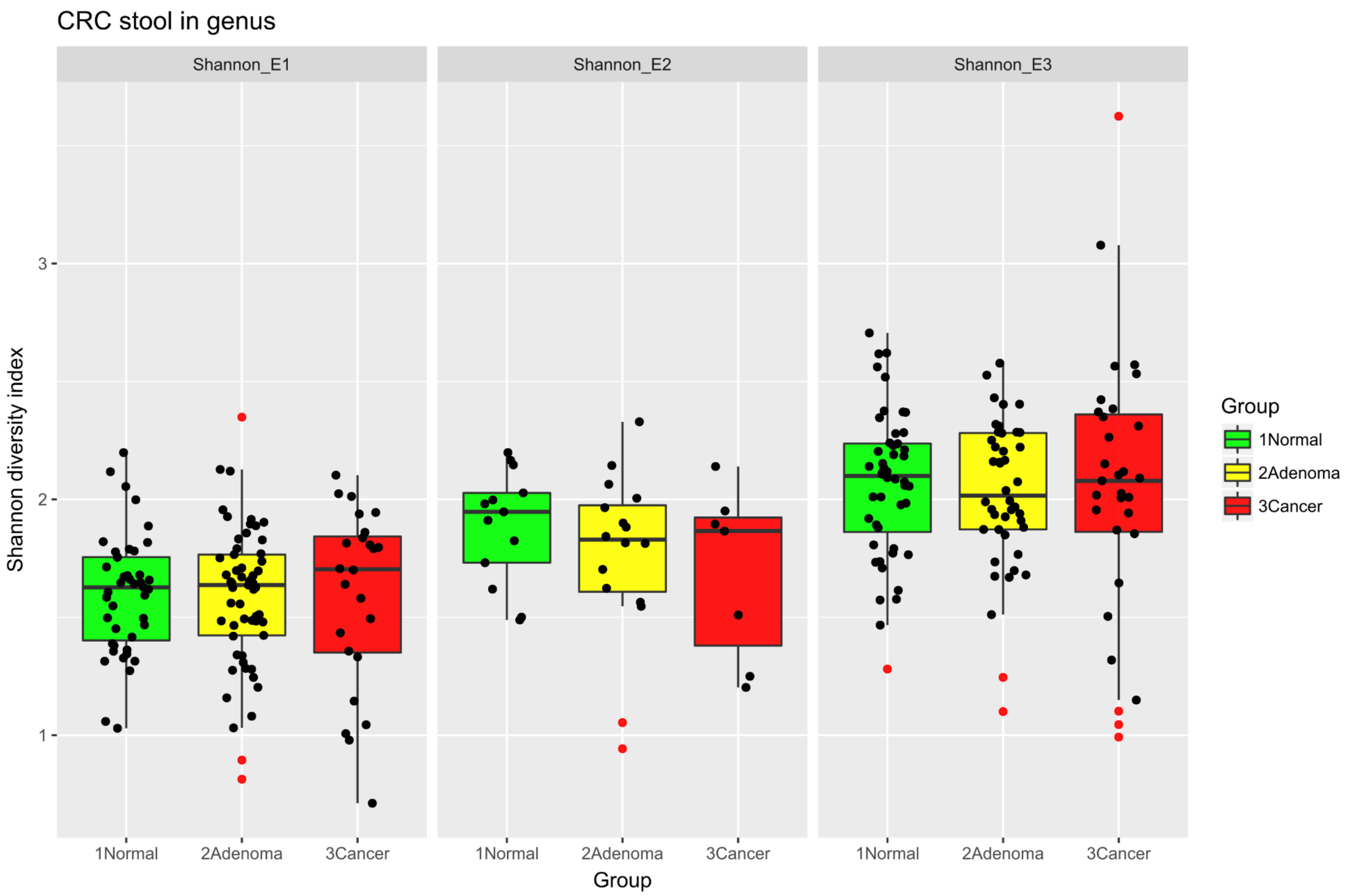

Figure S1A. Shannon diversity index by group across enterotypes Shannon diversity index of each sample in three enterotypes. Enterotype I & II have similar Shannon diversity index in each group. Enterotypes III has slightly higher Shannon diversity index, and the cancer group also varies slightly more without significance.

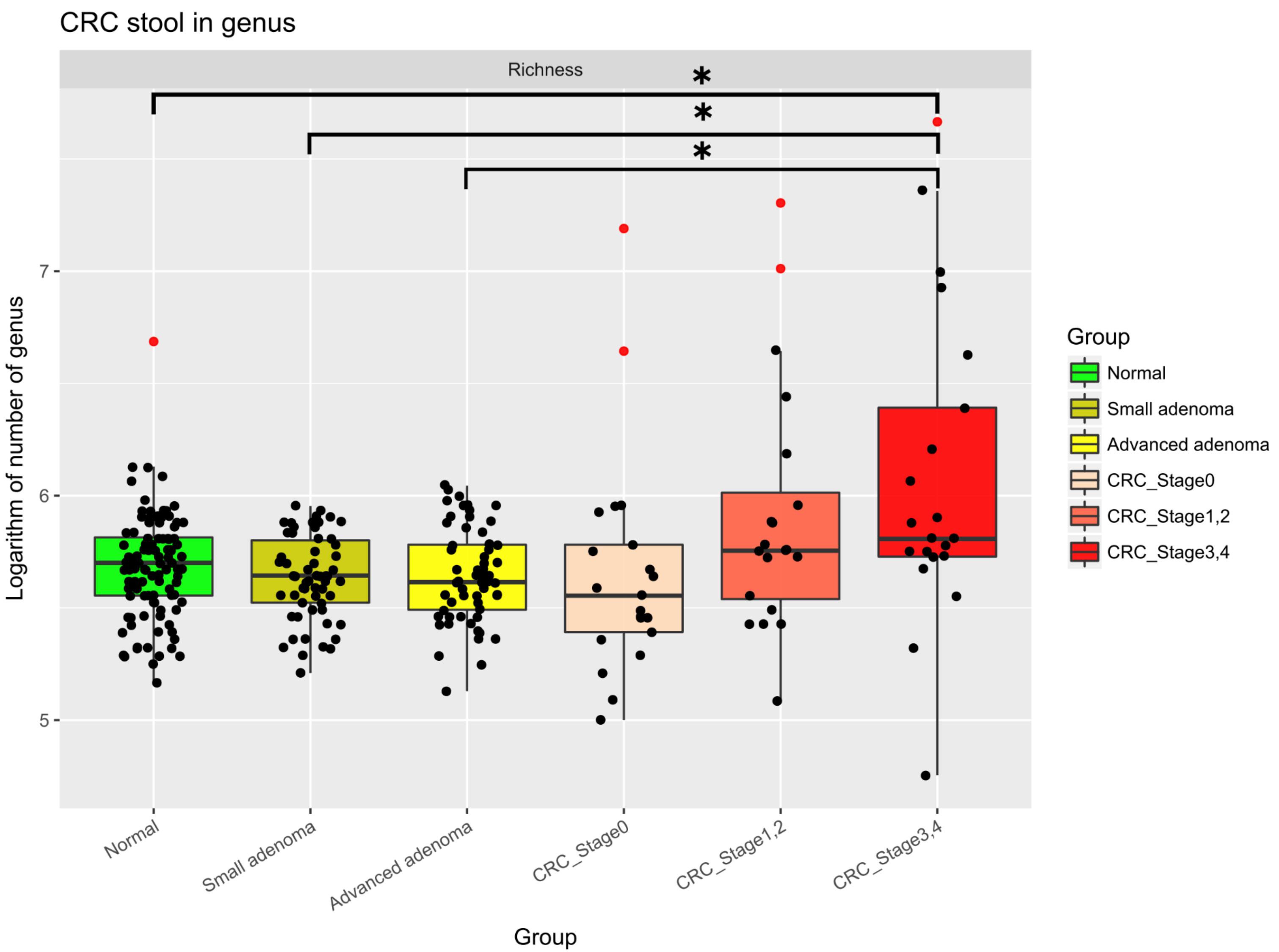

Figure S1B. Richness index by sub-group in all faecal samples Binary logarithm of genus richness of each sample in six sub-groups. The late stage cancer group has higher and varies more of richness significantly comparing to normal and adenoma groups. (* $p < 0.01$)

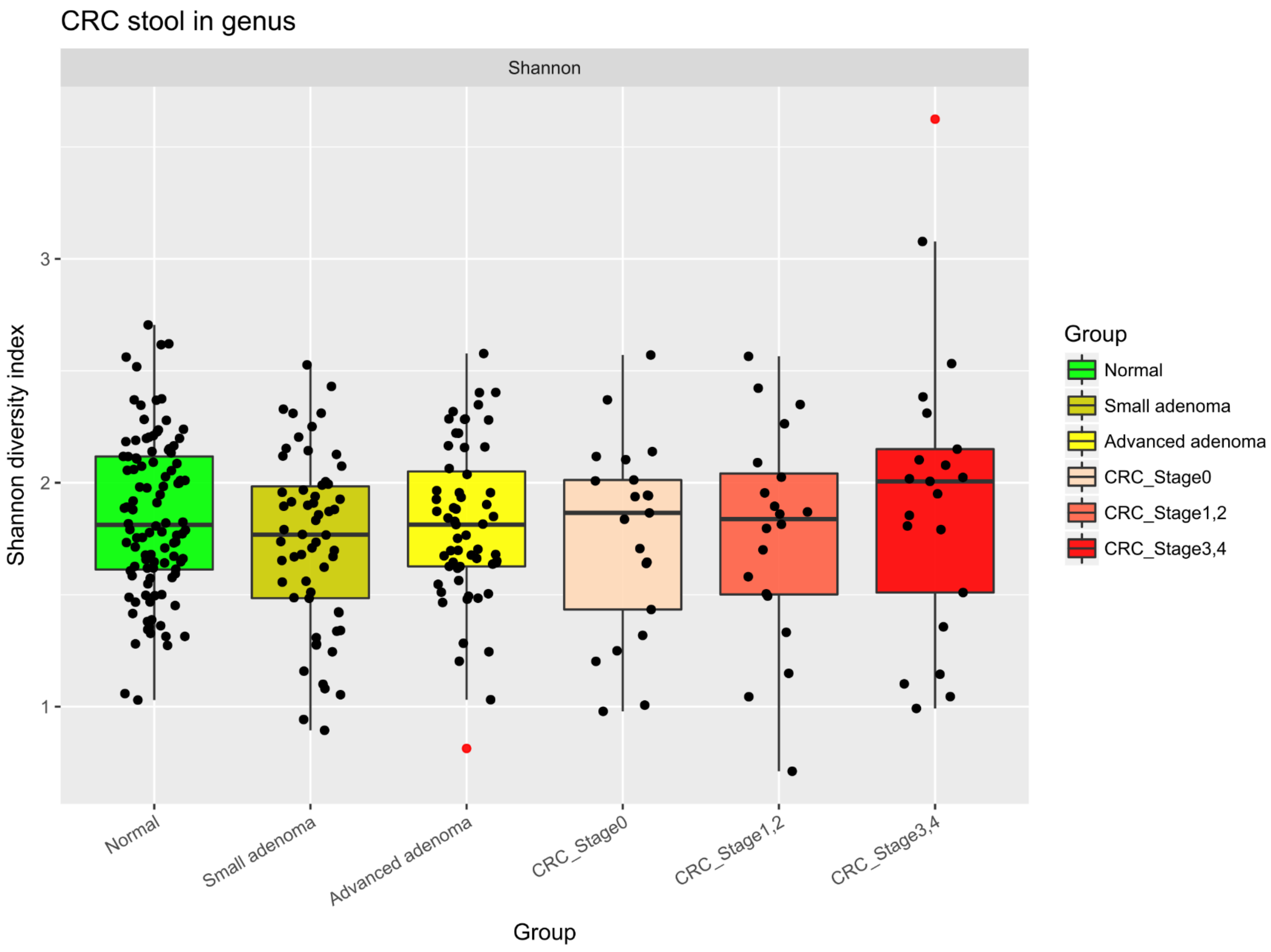

Figure S1C. Shannon diversity index by sub-group in all faecal samples Shannon diversity index of genus of each sample in six sub-groups. The late stage cancer group has slightly higher and varies more of Shannon diversity index but not significantly.

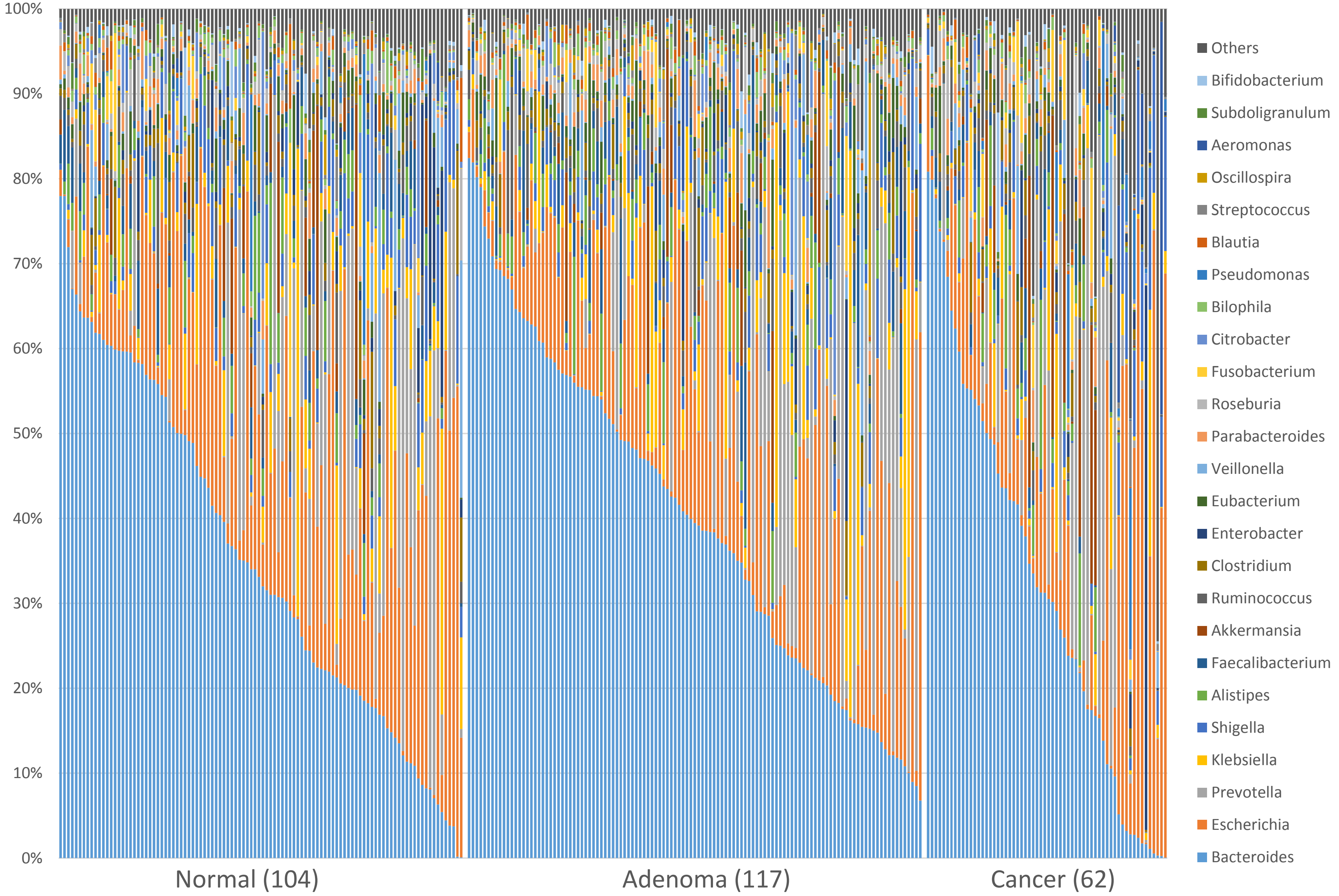

Figure S2A. Overall Microbial flora in genus level Relative abundance of top 25 genera of each subject grouped with normal, adenoma, and cancer, and sorted by *Bacteroides.* The composition of gut microbes in each subject varies across the groups while each group has similar diversity.

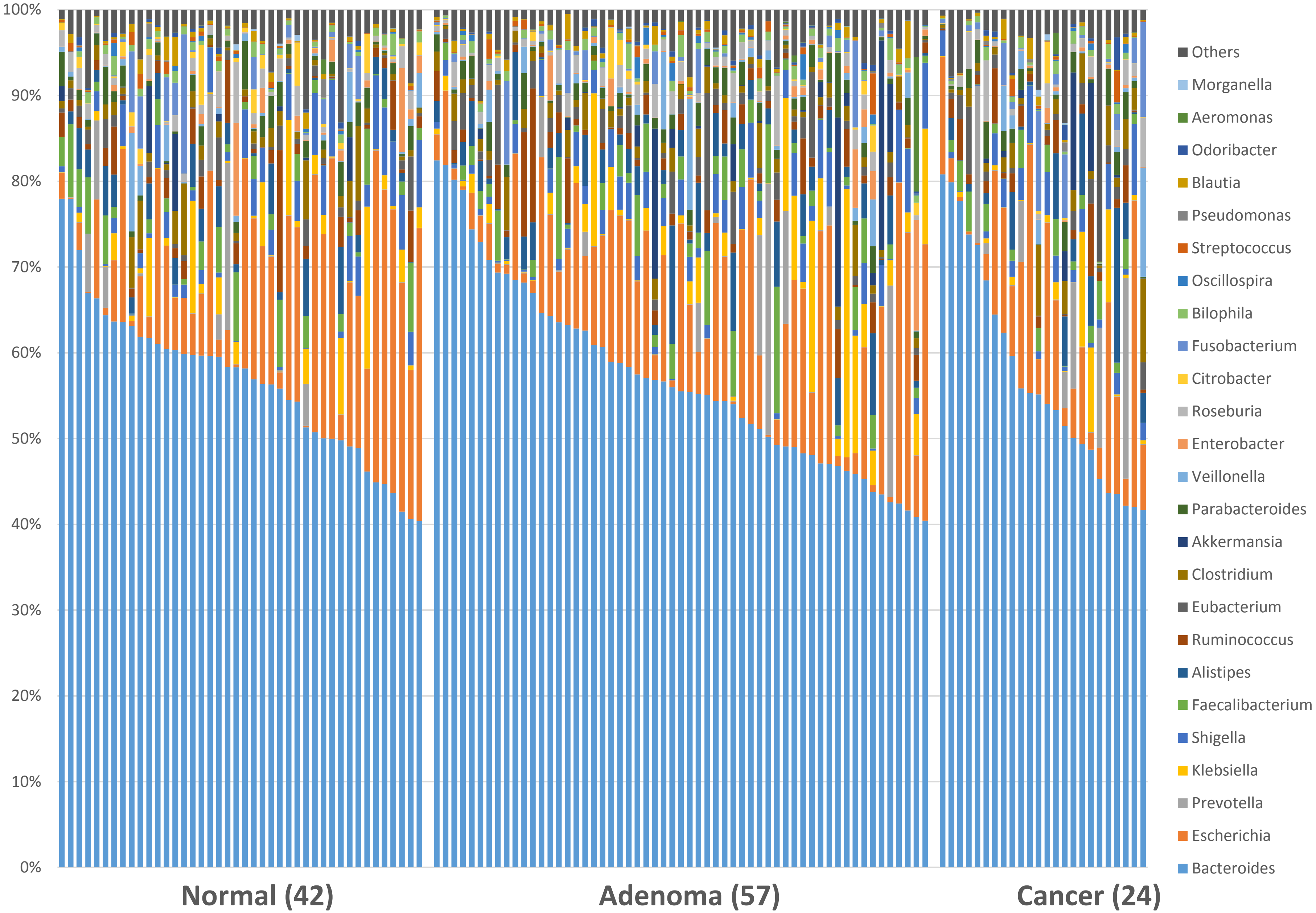

Figure S2B. Microbial flora of Enterotype I (*Bacteroides*-dominated) in genus level Relative abundance of top 25 genera of each subject in enterotypes I, grouped with normal, adenoma, and cancer, and sorted by *Bacteroides.* The portion of *Bacteroides* in each subject varies from 40% up to around 80% .

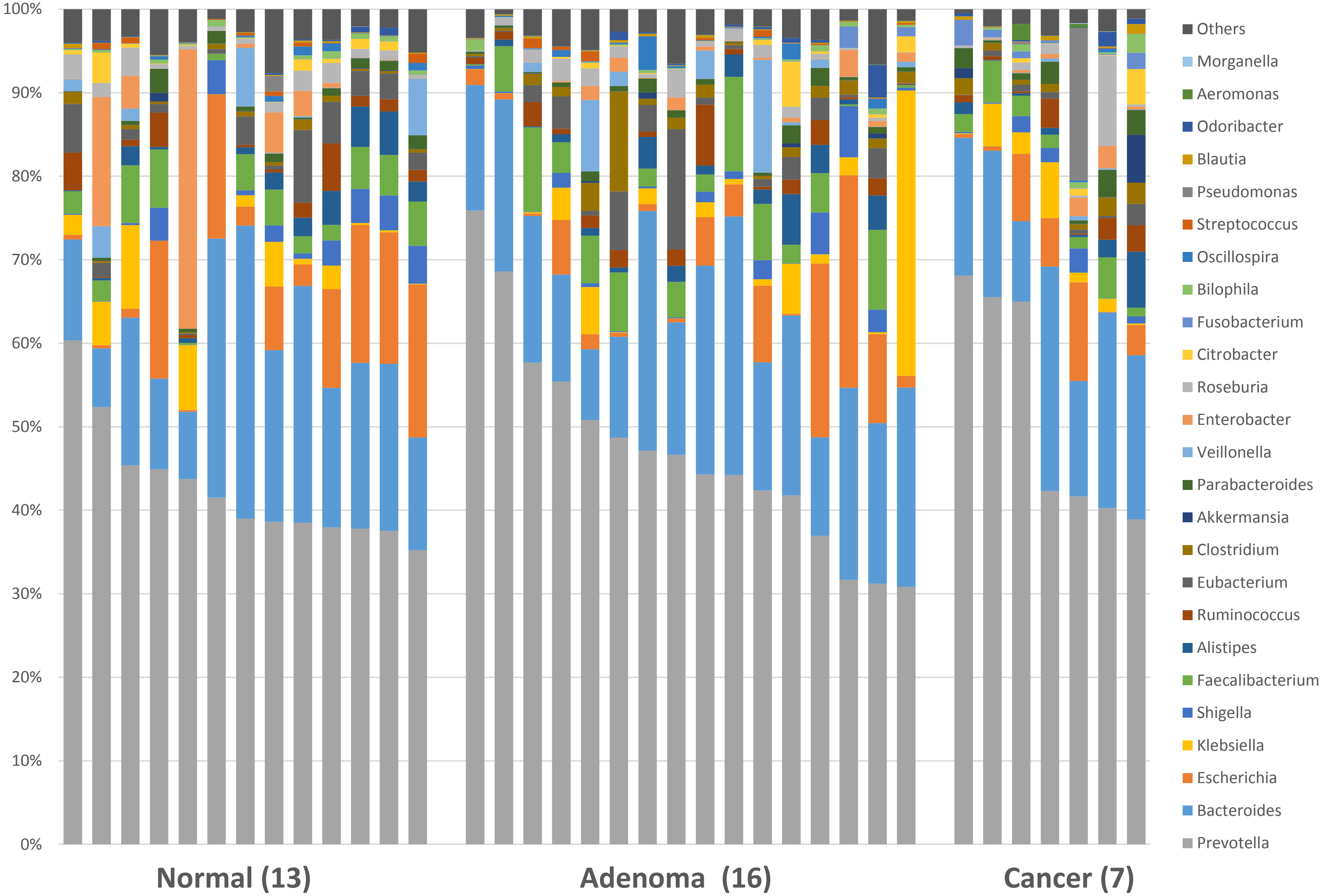

Figure S2C. Microbial flora of Enterotype II (*Prevotella*-dominated) in genus level Relative abundance of top 25 genera of each subject in enterotypes II, grouped with normal, adenoma, and cancer, and sorted by *Prevotella.* The portion of *Prevotella* in each subject varies from 30% up to around 75% .

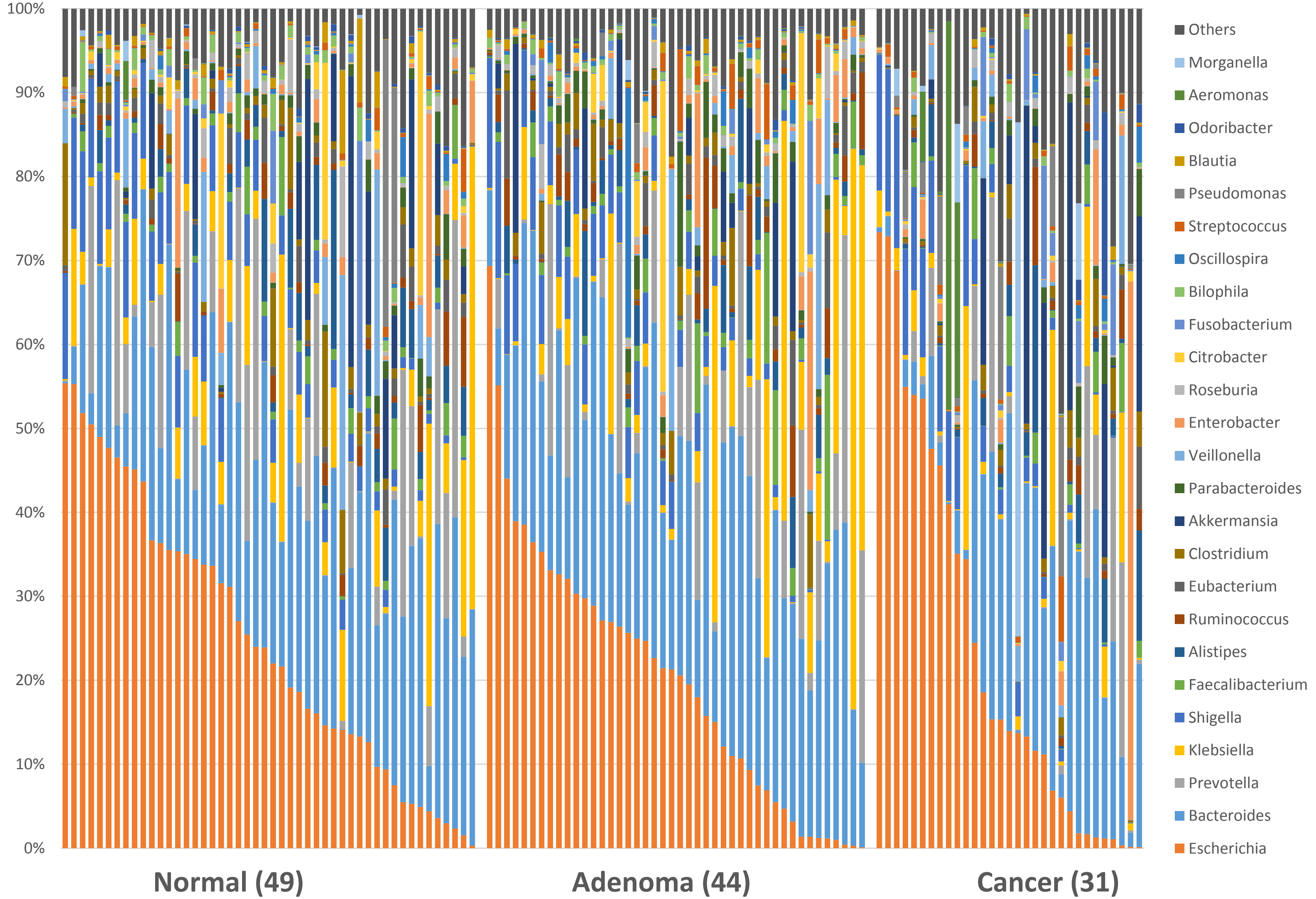

Figure S2D. Microbial flora of Enterotype III (*Escherichiaa*-dominated) in genus level Relative abundance of top 25 genera of each subject in enterotypes III, grouped with normal, adenoma, and cancer, and sorted by *Escherichia.* The portion of *Escherichia* in each subject varies up to around 70% .

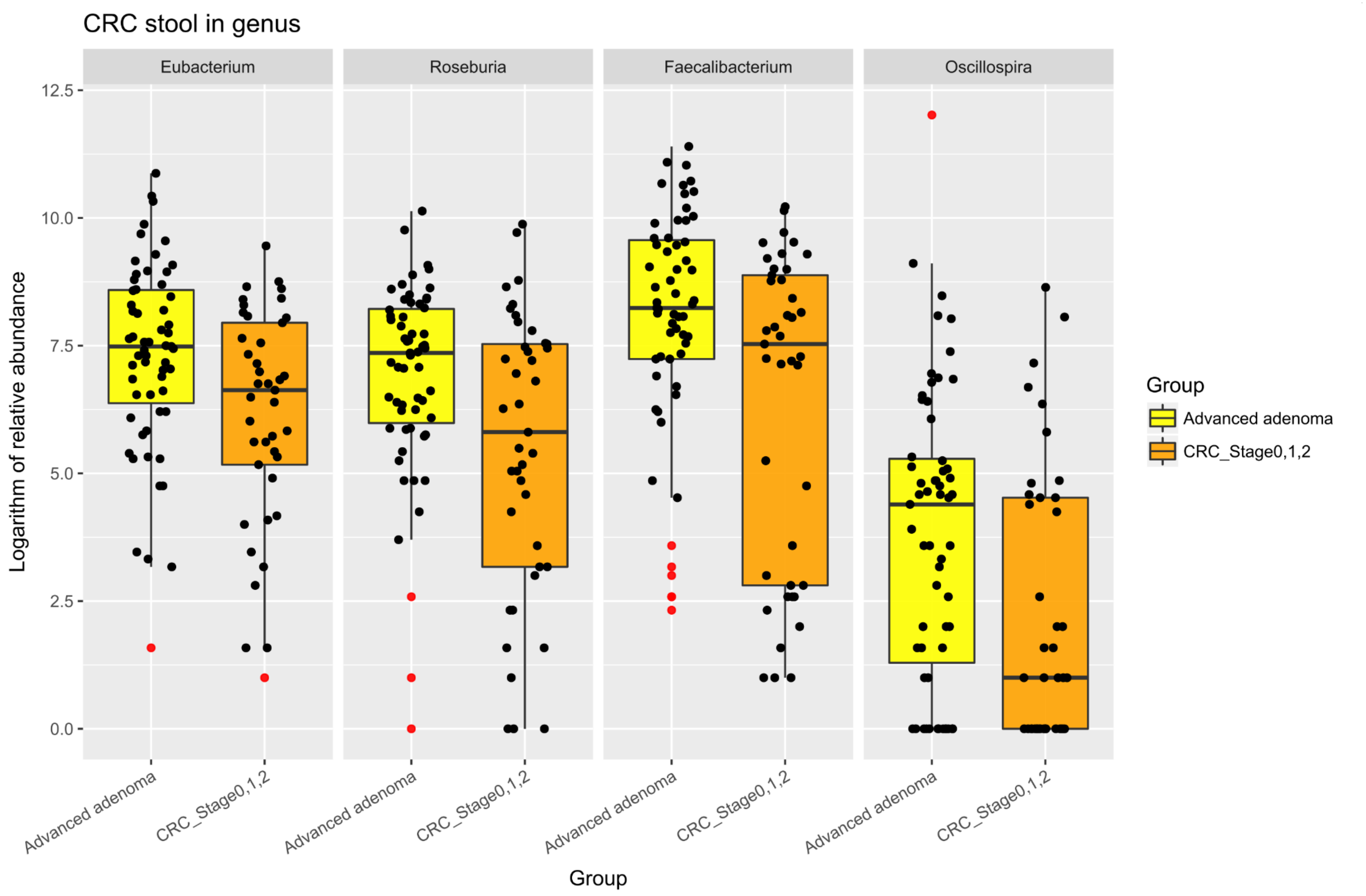

Figure S3. The relative abundance between advanced adenoma and early stage cancer Binary logarithm of relative abundance of single genus in two sub-groups. The relative abundance of *Eubacterium, Roseburia, Faecalibacterium*, and *Oscillospira* in CRC stage 0,1,2 group are significantly lower than in advanced adenoma group. Each dot represented one sample, outlier samples were marked as red dot. ($p < 0.01$)

NC correlated

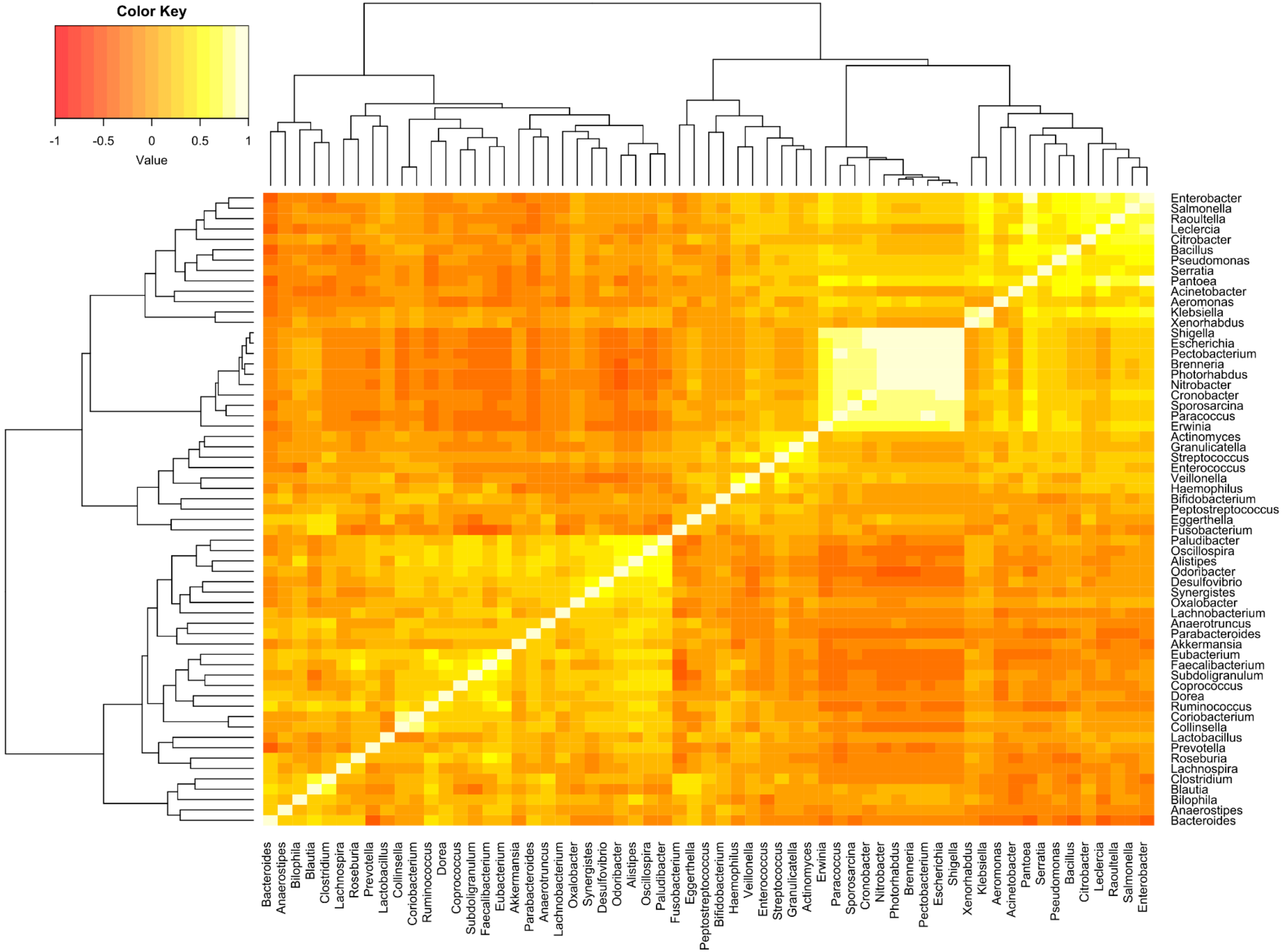

Figure S4A. Spearman's correlation analysis of normals and CRCs Genera appearance greater than 50% in cancer group were included to calculate the correlation coefficient by relative abundance. Only microflora of normals and CRCs were employed.

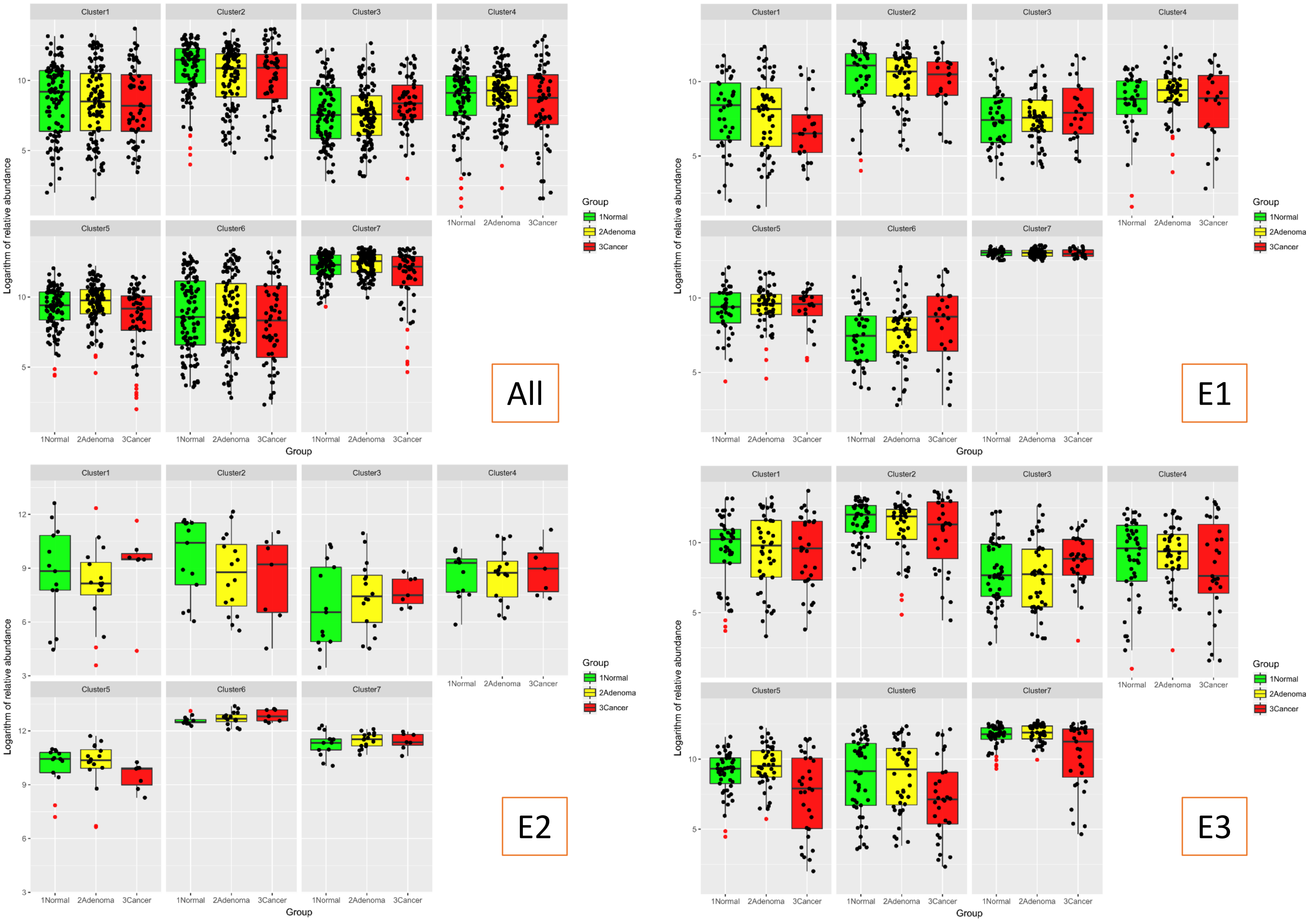

Figure S4B. Boxplots of relative abundance of CAGs clustered by normals and CRCs Summation of relative abundance of 7 NC correlated CAGs were illustrated based on all samples and samples of each enterotype.

NAC correlated

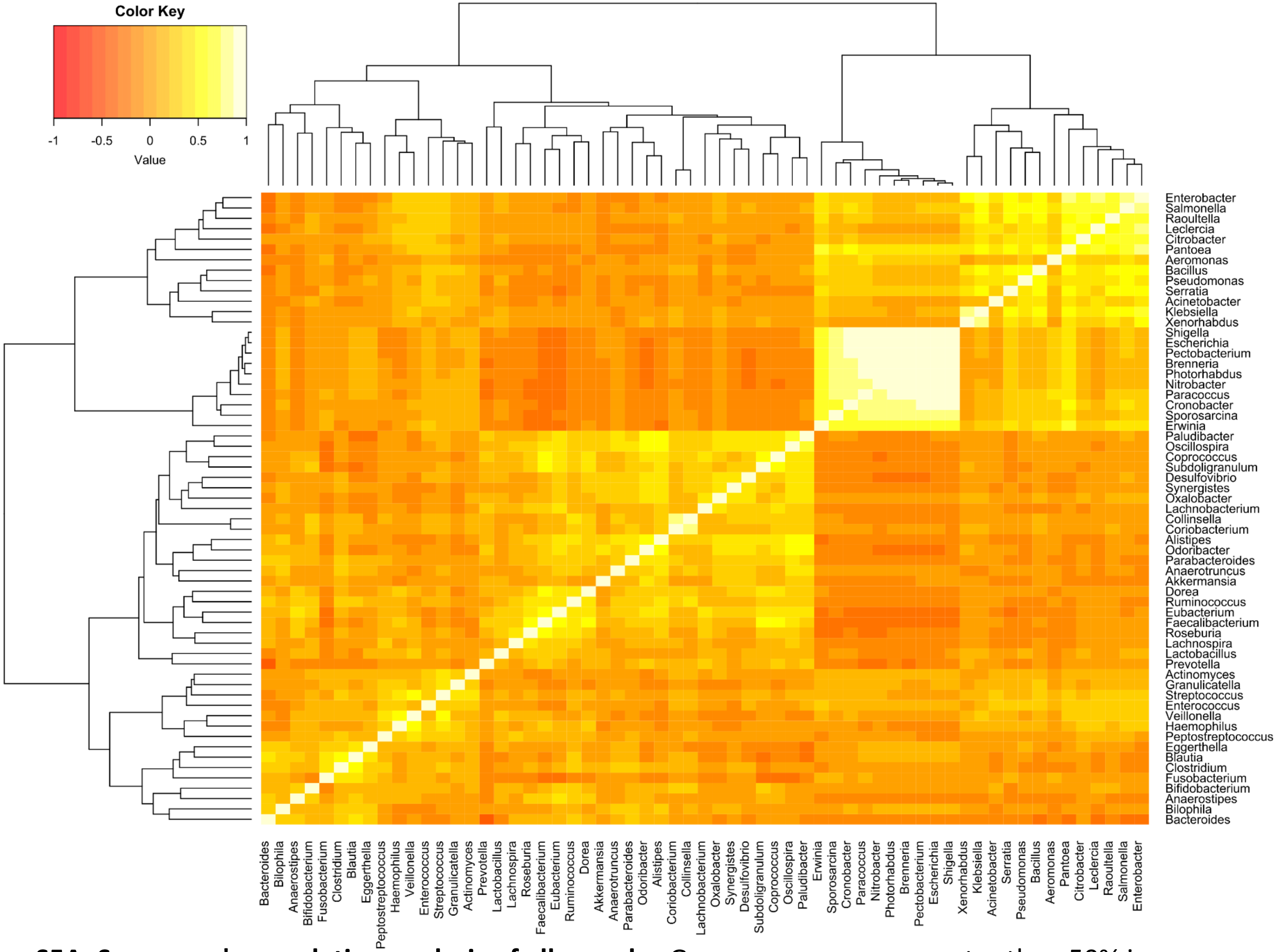

Figure S5A. Spearman's correlation analysis of all samples Genera appearance greater than 50% in cancer group were included to calculate the correlation coefficient by relative abundance. Microflora of all samples were employed.

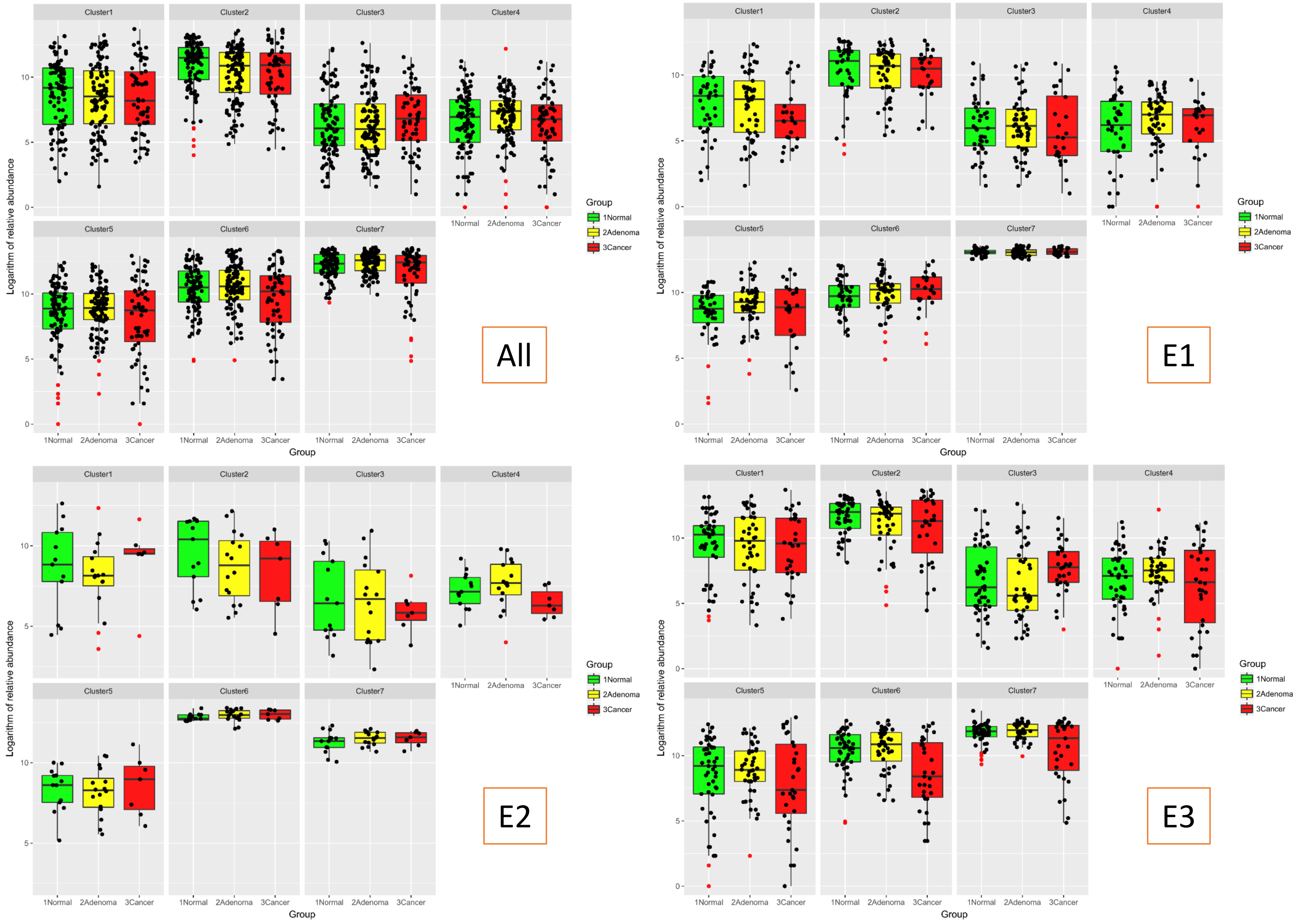

Figure S5B. Boxplots of relative abundance of CAGs clustered by all samples Summation of relative abundance of 7 NAC correlated CAGs were illustrated based on all samples and samples of each enterotype.

NAC correlated

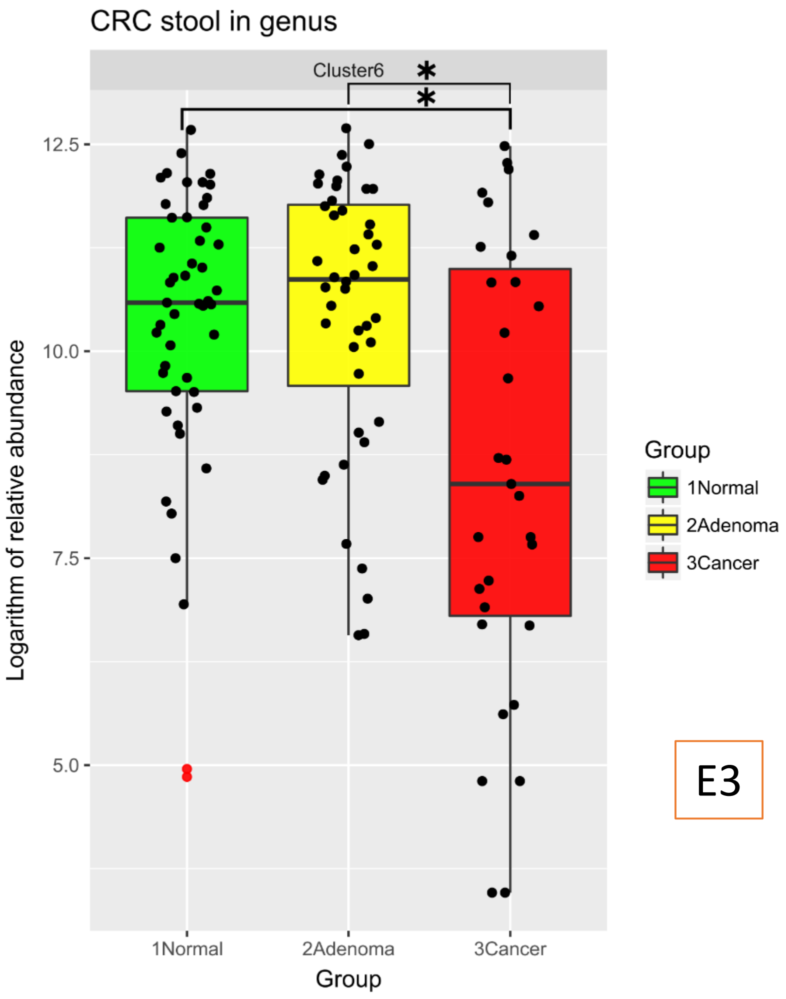

E3

Figure S5C. Boxplots of relative abundance of significant CAG Summation of relative abundance of NAC correlated cluster 6 showed significant lower abundance in CRC group than normal and adenoma groups.

NA correlated

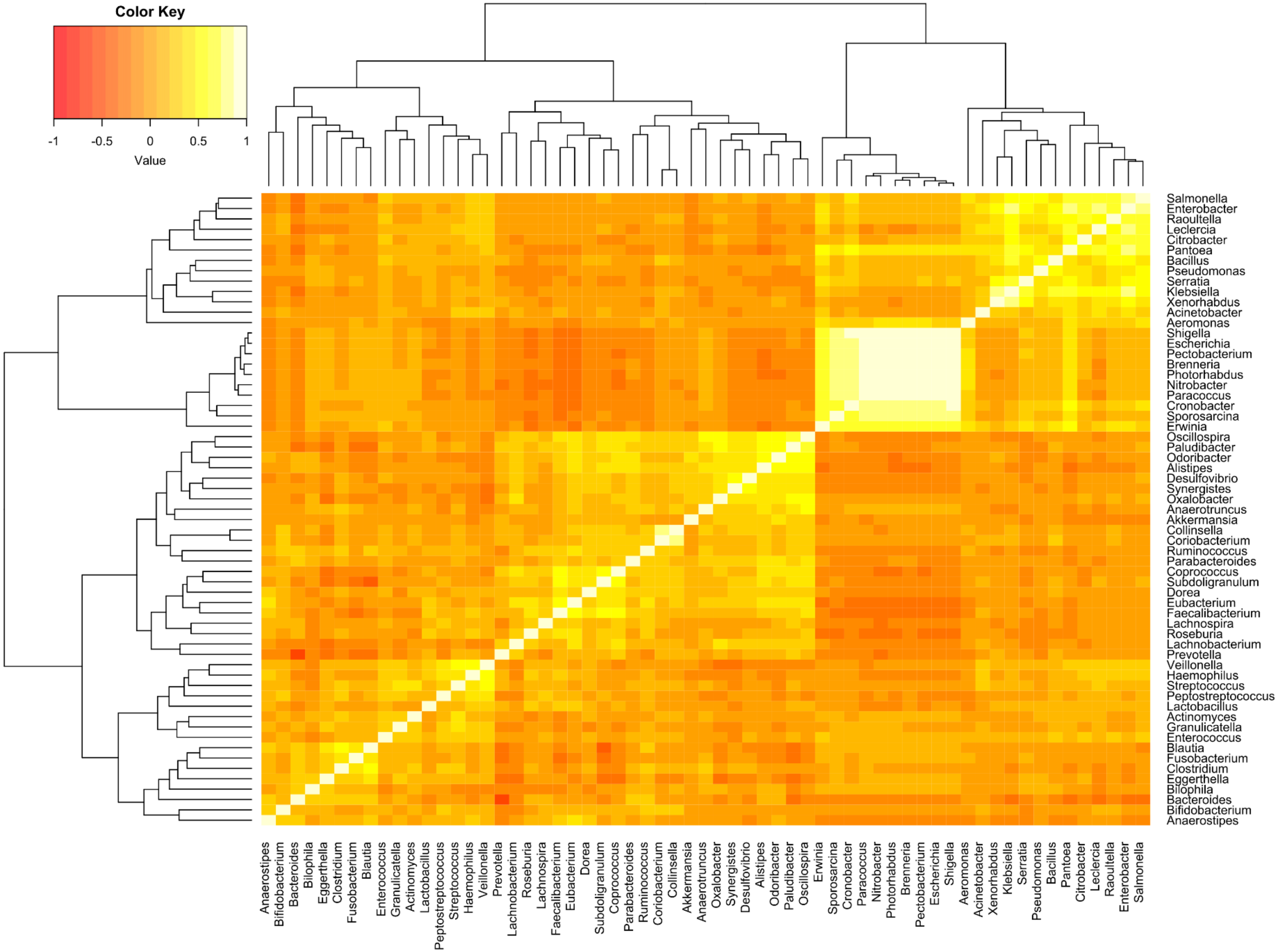

Figure S6A. Spearman's correlation analysis of normals and adenomatous polyps Genera appearance greater than 50% in cancer group were included to calculate the correlation coefficient by relative abundance. Only microflora of normals and adenomatous polyps were employed.

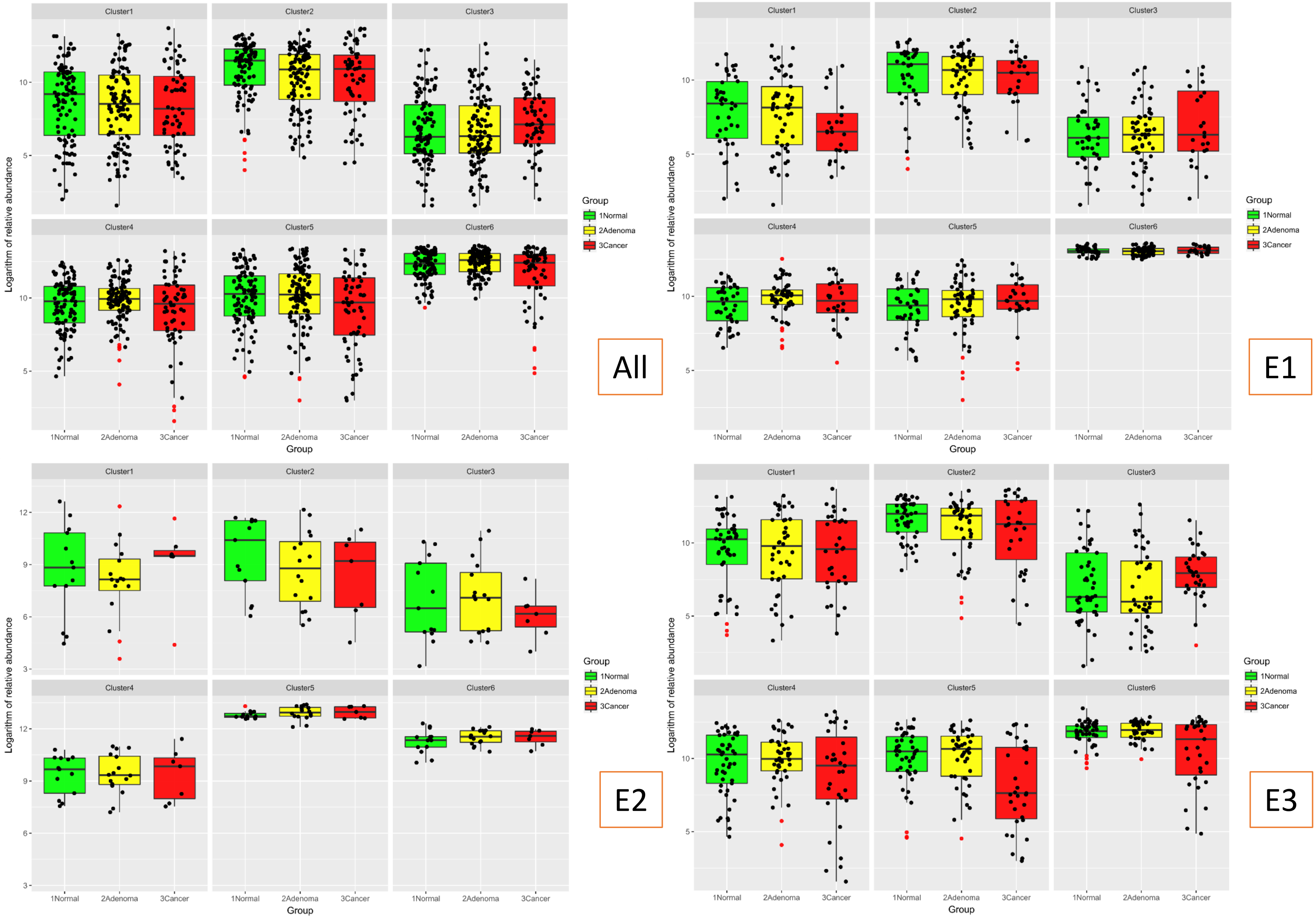

Figure S6B. Boxplots of relative abundance of CAGs clustered by normals and adenomatous polyps Summation of relative abundance of 6 NA correlated CAGs were illustrated based on all samples and samples of each enterotype.

NA correlated

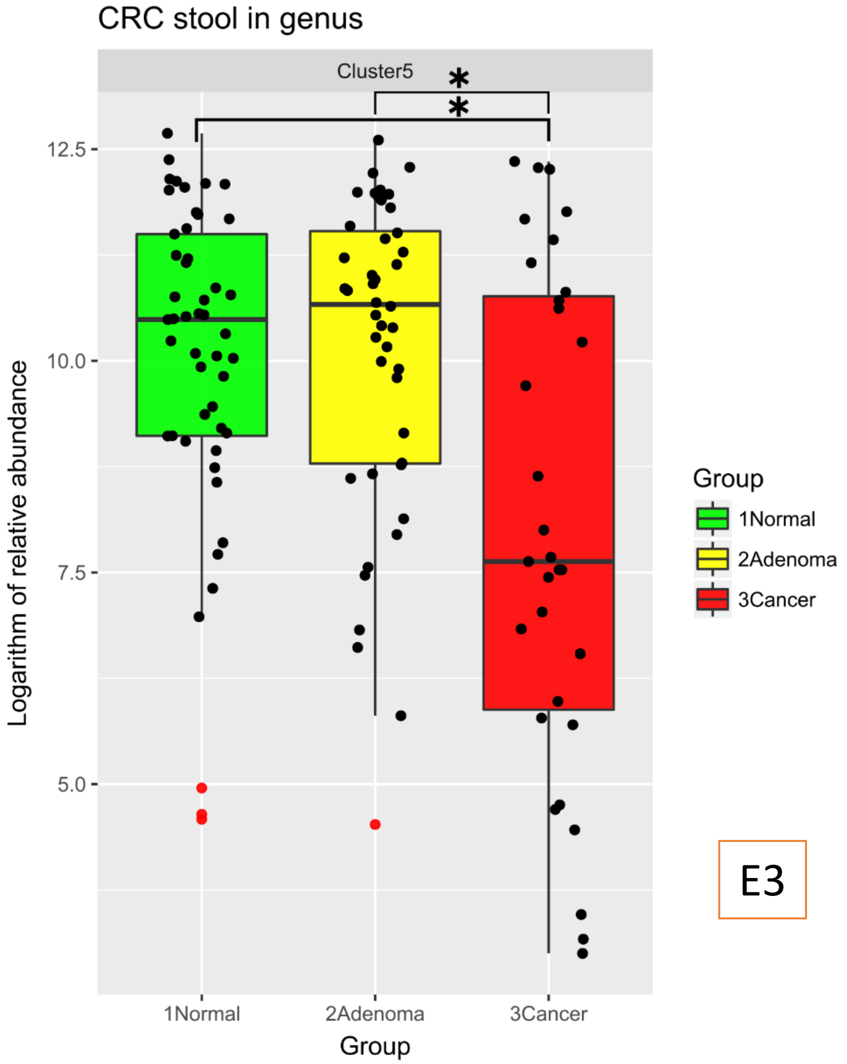

Figure S6C. Boxplots of relative abundance of significant CAG Summation of relative abundance of NA correlated cluster 5 showed significant lower abundance in CRC group than normal and adenoma groups.

AC correlated

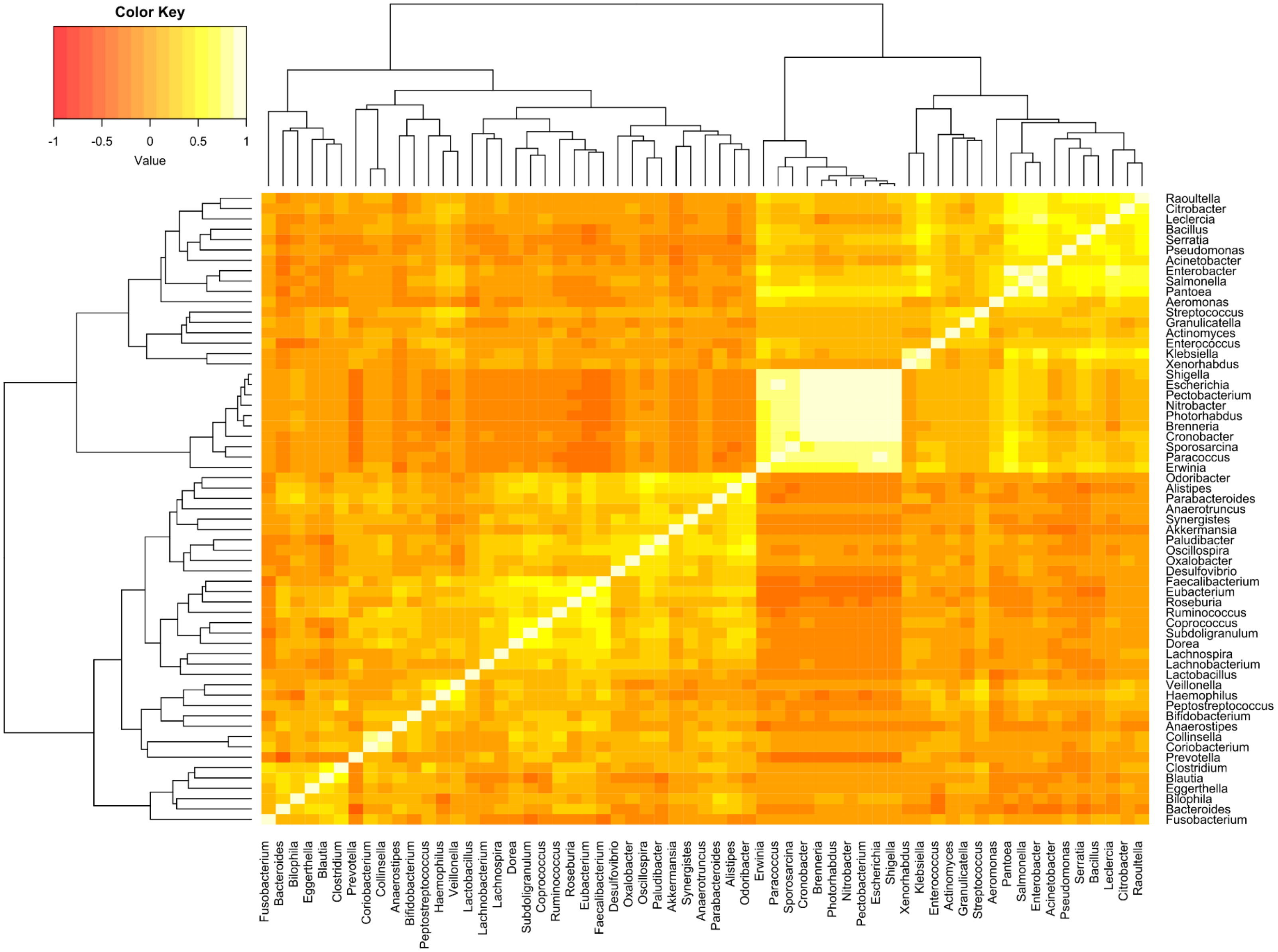

Figure S7A. Spearman's correlation analysis of adenomatous polyps and CRCs Genera appearance greater than 50% in cancer group were included to calculate the correlation coefficient by relative abundance. Only microflora of adenomatous polyps and CRCs were employed.

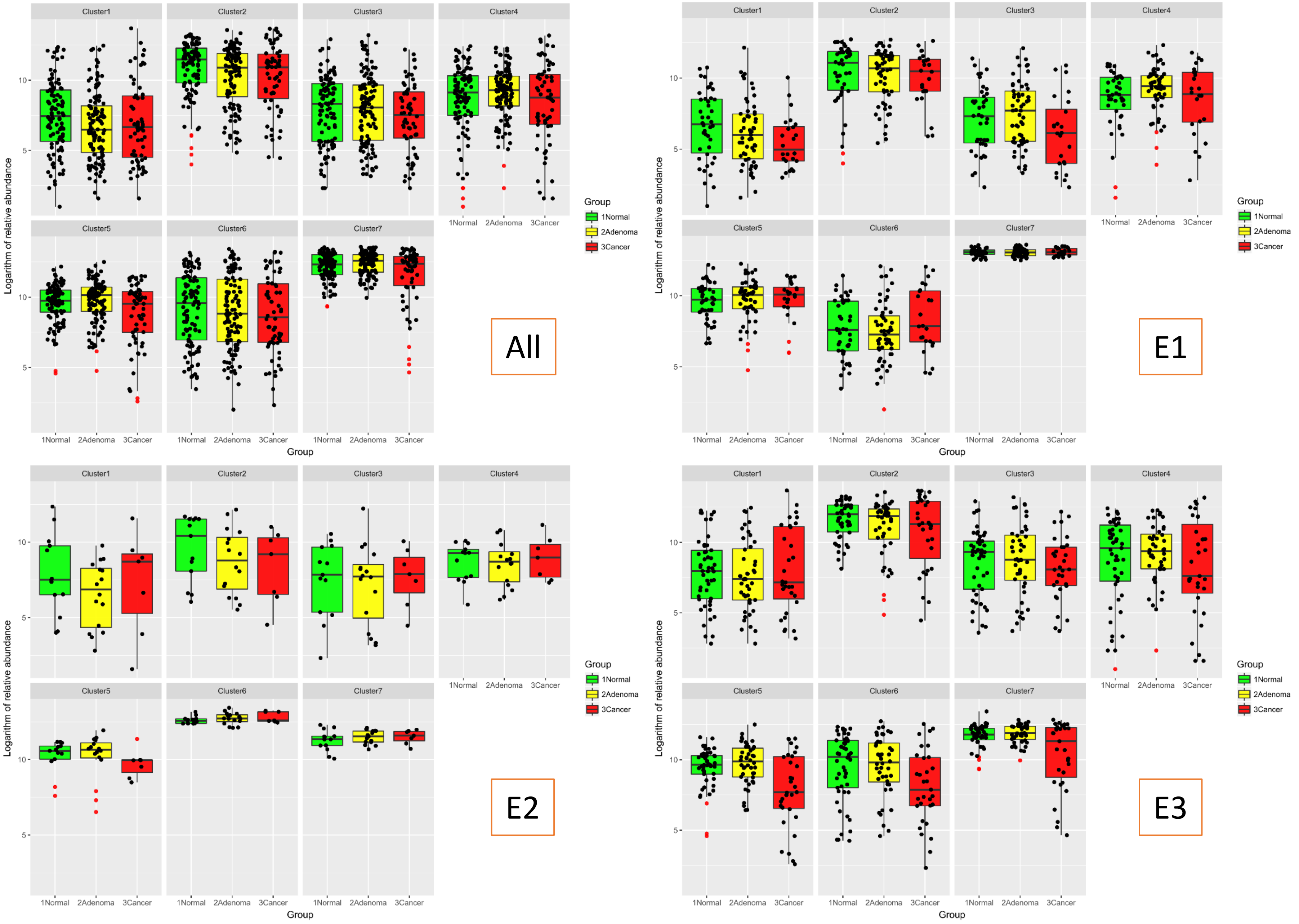

Figure S7B. Boxplots of relative abundance of CAGs clustered by adenomatous polyps and CRCs Summation of relative abundance of 7 NC correlated CAGs were illustrated based on all samples and samples of each enterotype.

AC correlated

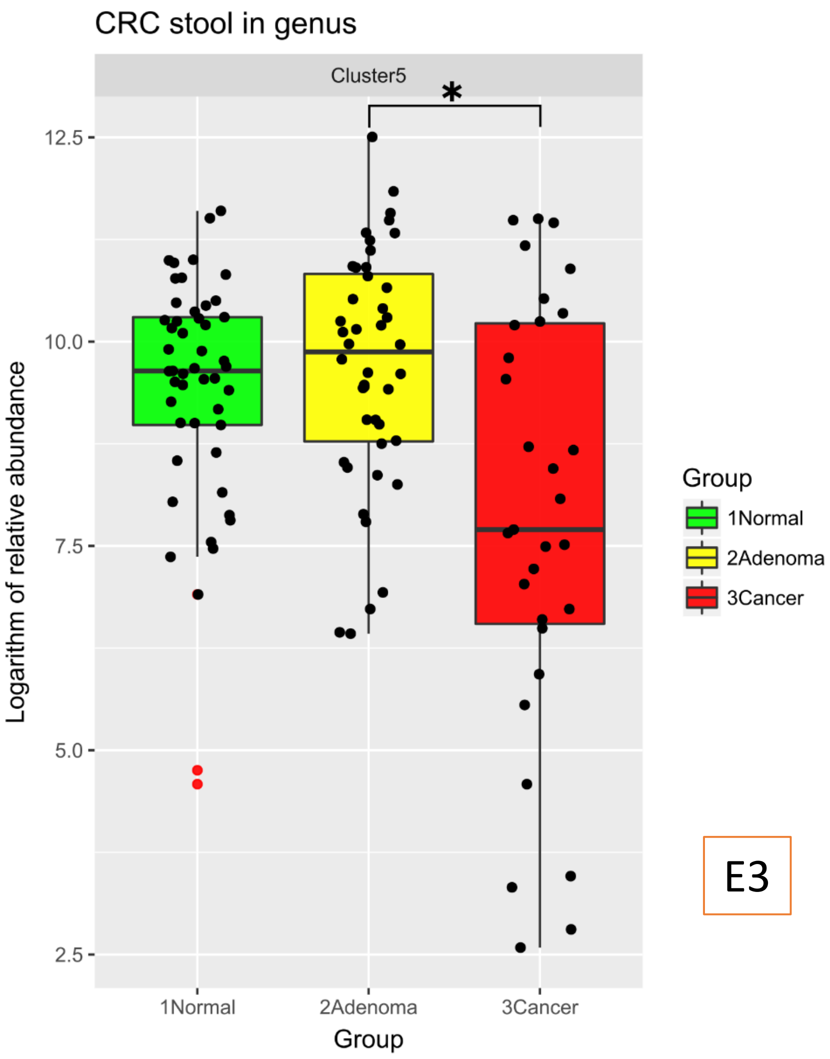

E3

Figure S7C. Boxplots of relative abundance of significant CAG Summation of relative abundance of AC correlated cluster 5 showed significant lower abundance in CRC group than adenoma groups.

Enterotype I - *Bacteroides* dominated

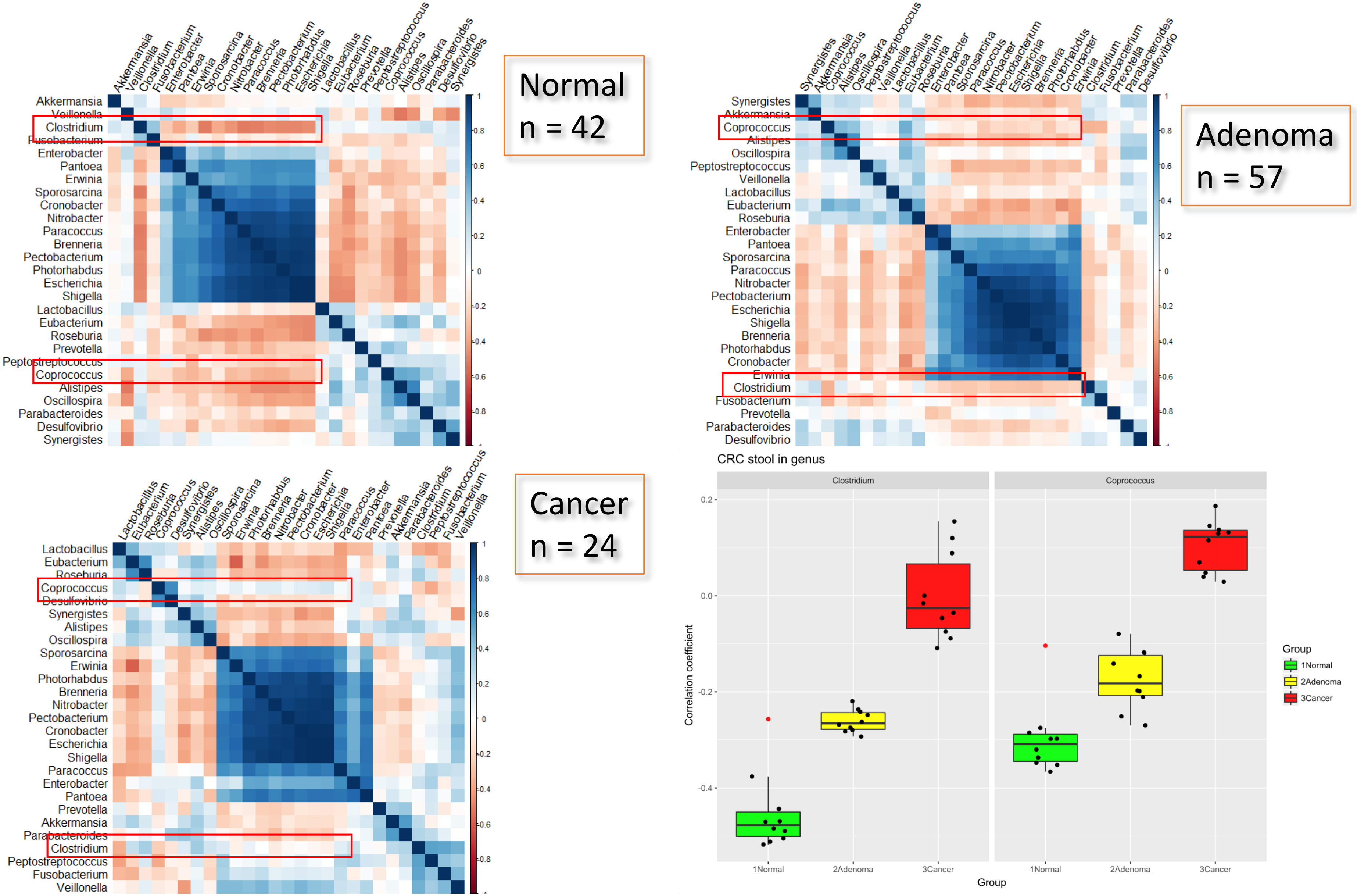

Figure S8A. Using the steady CAG (cluster 2) as a standard to identify specific genus that differed between groups – Enterotype I The correlation coefficient of *Clostridium* and *Coprococcus* with cluster 2 varied in normal, adenoma, and cancer groups in enterotypes 1.

Enterotype II - *Prevotella* dominated

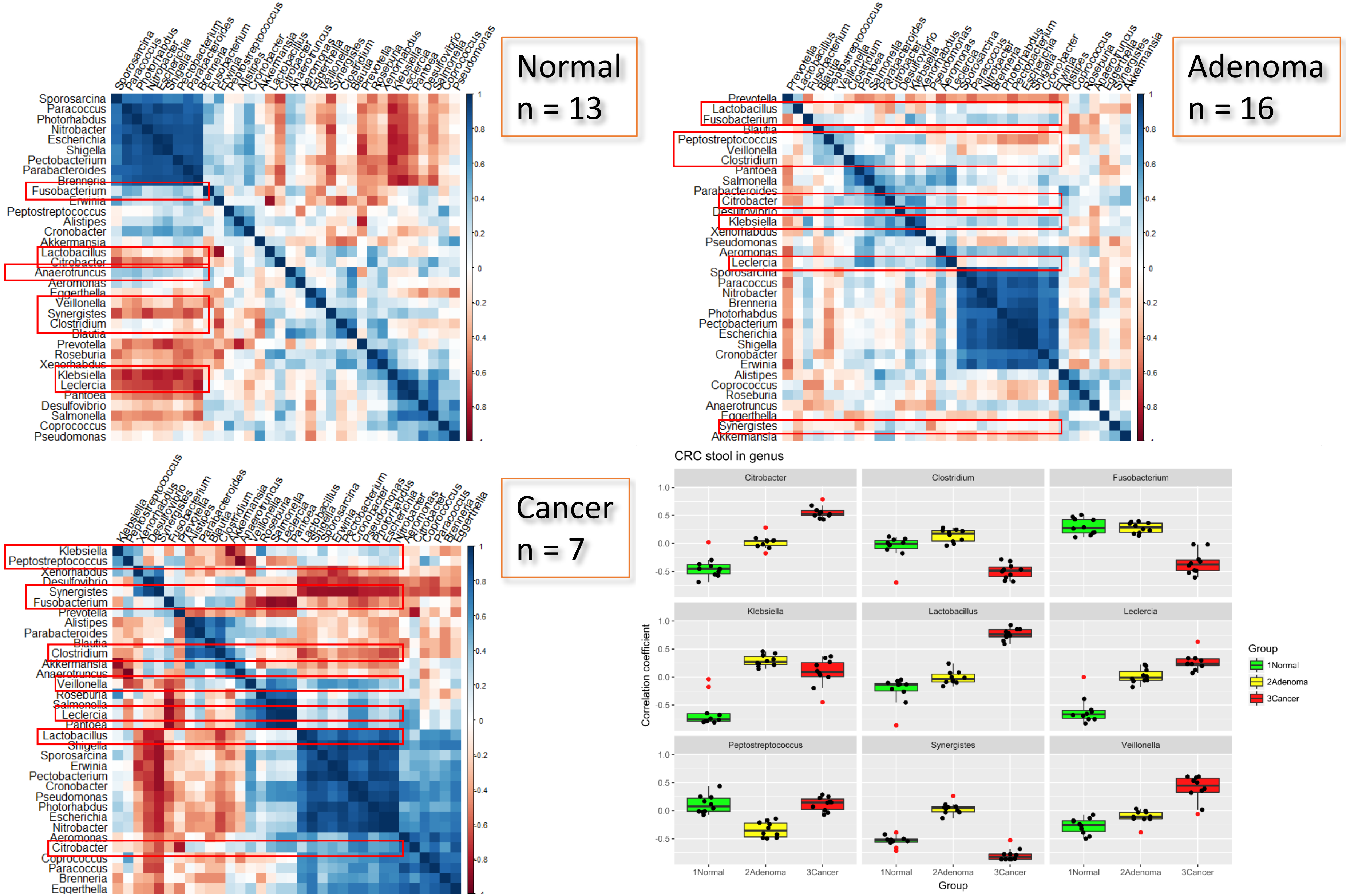

Figure S8B. Using the steady CAG (cluster 2) as a standard to identify specific genus that differed between groups – Enterotype II The correlation coefficient of 9 genera with cluster 2 varied in normal, adenoma, and cancer groups in enterotypes 2.

Enterotype III - *Escherichia* dominated

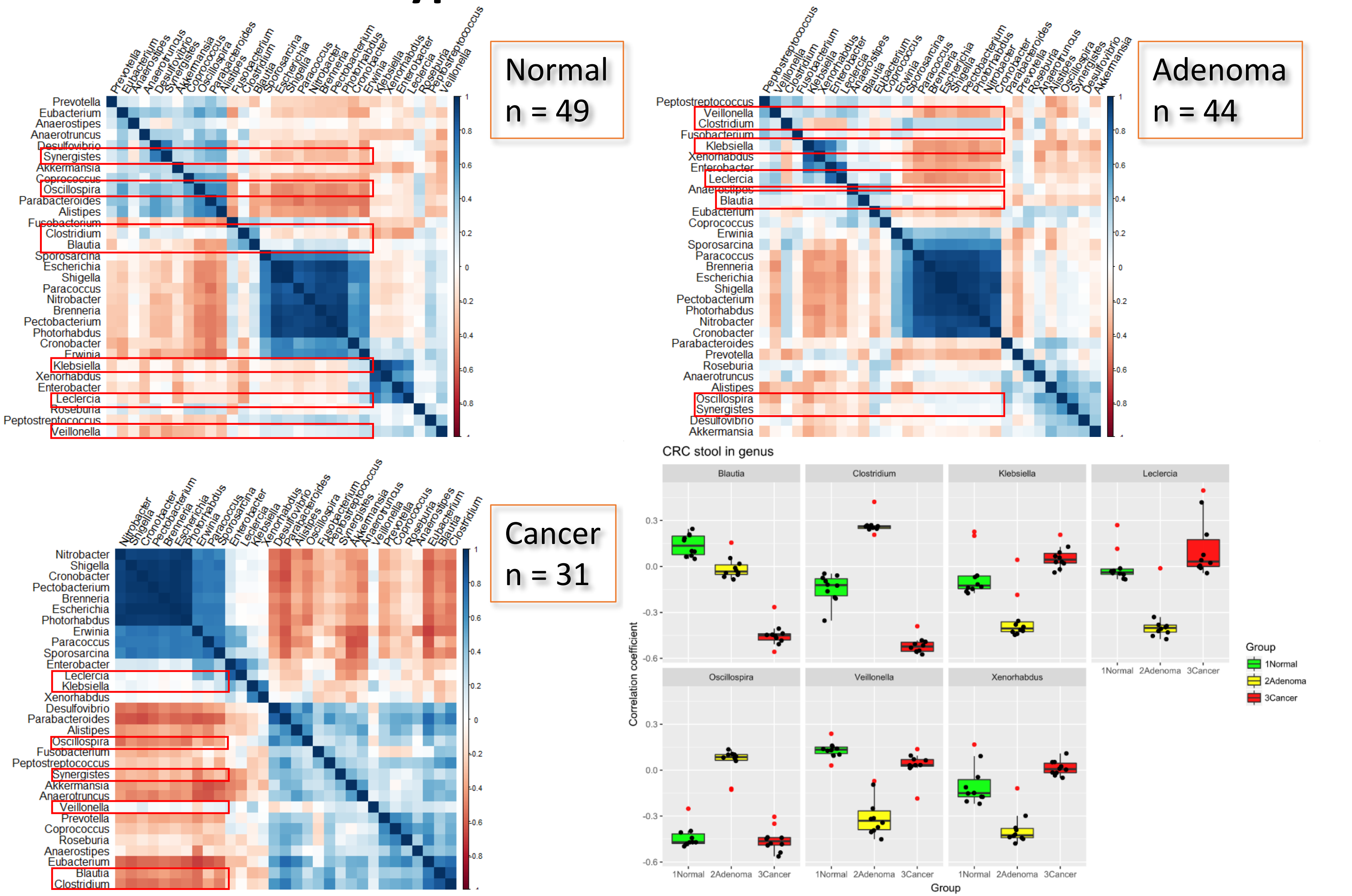

Figure S8C. Using the steady CAG (cluster 2) as a standard to identify specific genus that differed between groups – Enterotype III The correlation coefficient of 7 genera with cluster 2 varied in normal, adenoma, and cancer groups in enterotypes 3.

Correlation network – Enterotype II

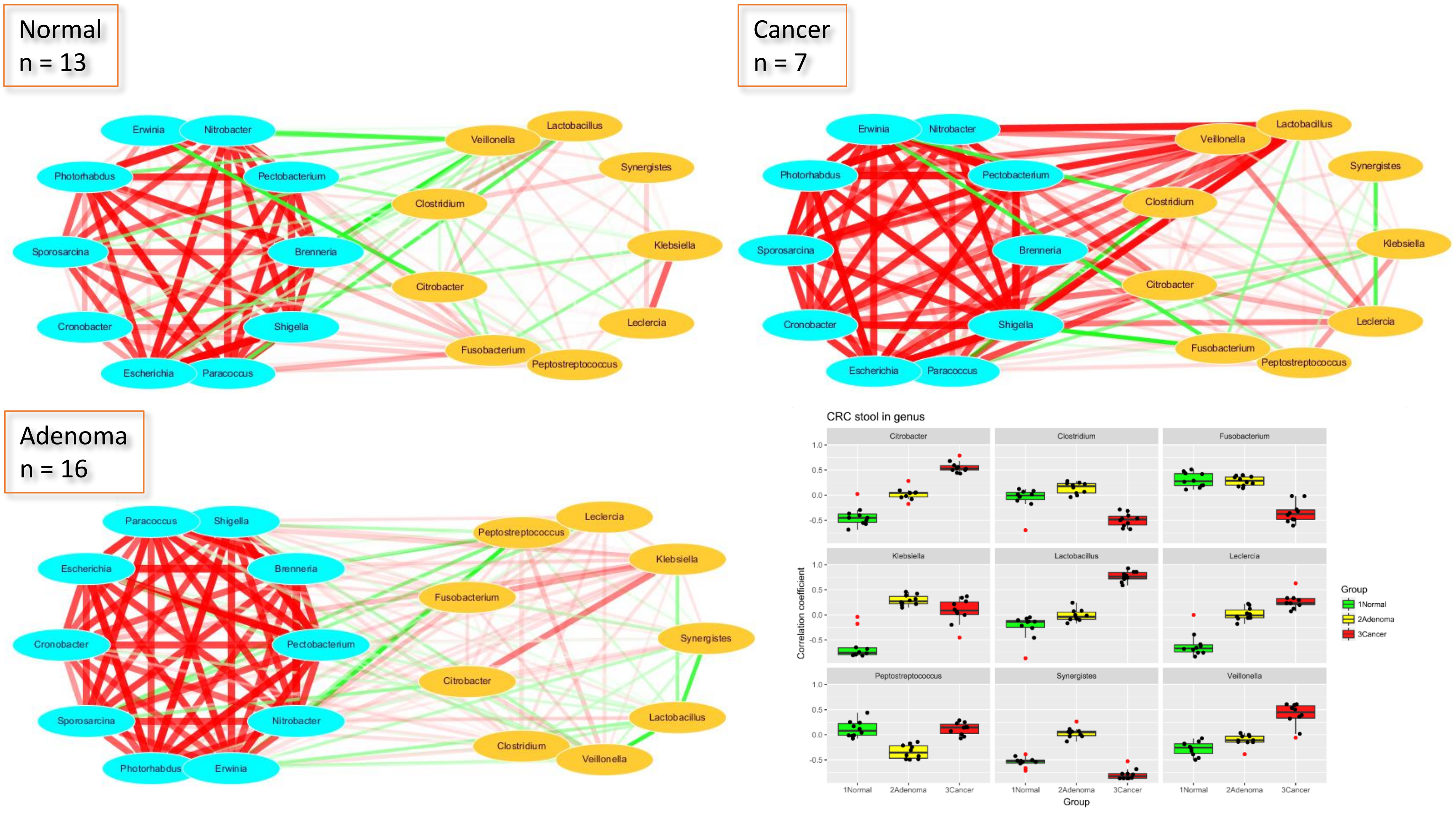

Figure S9. Network analysis of stool microbiota using Pearson's correlation coefficients (Enterotype II) Correlation coefficients network between 10 genera of CAG 2 and 9 other genera.

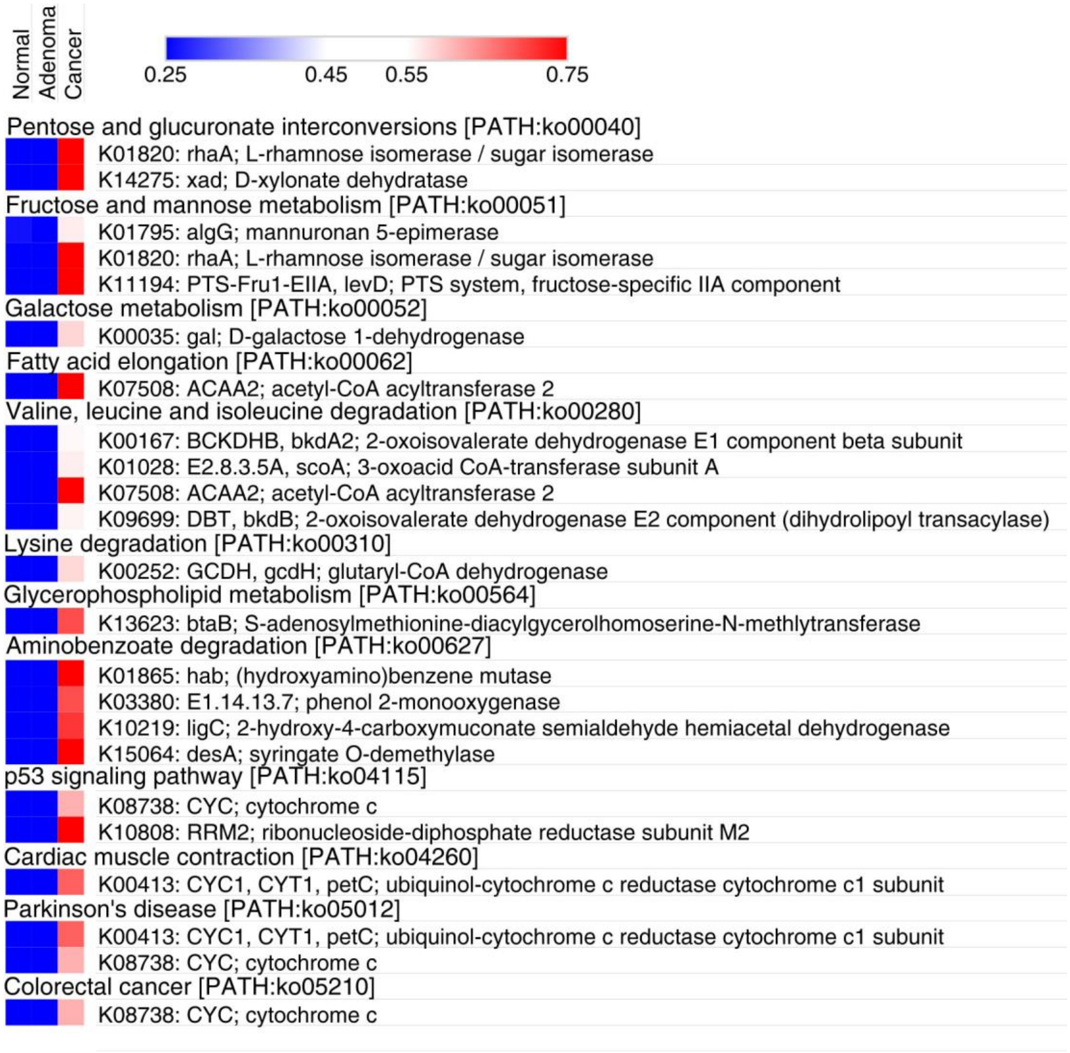

Figure S10A. Functional analysis of stool microbiota with a positive correlation of CRC.

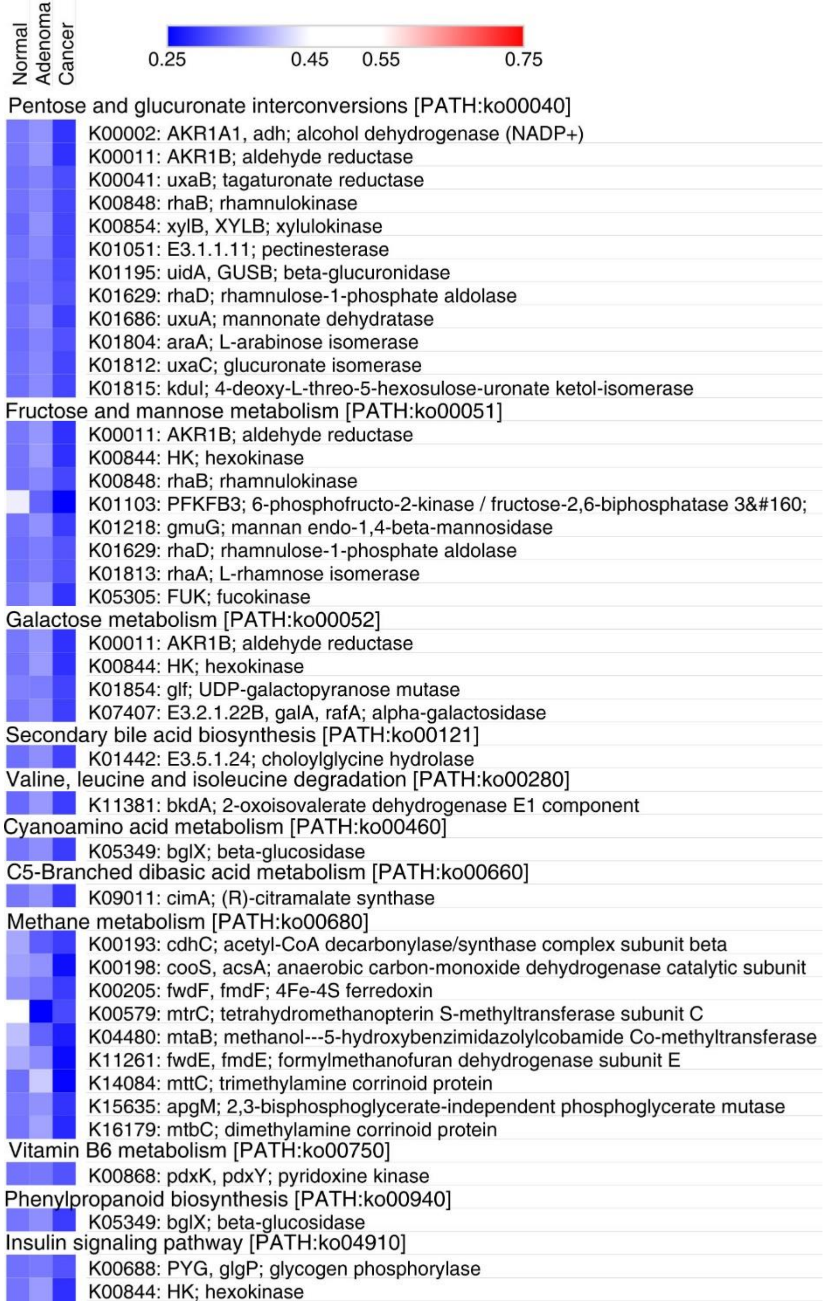

Figure S10B. Functional analysis of stool microbiota with a negative correlation of CRC.

Appendix. All primers used in this study

Oligo		Sequence (5'-3')
Nextera adapter + V3V4 Primer	Forward	5'-TCGTCGGCAGCGTCAGATGTGTATAAGAGACAGCCTACGGGNGGCWGCAG
	Reverse	5'-GTCTCGTGGGCTCGGAGATGTGTATAAGAGACAGGACTACHVGGGTATCTAATCC
Nextera Index Primer		
Index i5	S502	5'-AATGATACGGCGACCACCGAGATCTACACCTCTCTATTCGTCGGCAGCGTC
	S503	5'-AATGATACGGCGACCACCGAGATCTACACTATCCTCTTCGTCGGCAGCGTC
	S505	5'-AATGATACGGCGACCACCGAGATCTACACGTAAGGAGTCGTCGGCAGCGTC
	S506	5'-AATGATACGGCGACCACCGAGATCTACACACTGCATATCGTCGGCAGCGTC
	S507	5'-AATGATACGGCGACCACCGAGATCTACACAAGGAGTATCGTCGGCAGCGTC
	S508	5'-AATGATACGGCGACCACCGAGATCTACACCTAAGCCTTCGTCGGCAGCGTC
	S510	5'-AATGATACGGCGACCACCGAGATCTACACCGTCTAATTCGTCGGCAGCGTC
	S511	5'-AATGATACGGCGACCACCGAGATCTACACTCTCTCCGTCGTCGGCAGCGTC
	S513	5'-AATGATACGGCGACCACCGAGATCTACACTCGACTAGTCGTCGGCAGCGTC
	S515	5'-AATGATACGGCGACCACCGAGATCTACACTTCTAGCTTCGTCGGCAGCGTC
	S516	5'-AATGATACGGCGACCACCGAGATCTACACCCTAGAGTTCGTCGGCAGCGTC
	S517	5'-AATGATACGGCGACCACCGAGATCTACACGCGTAAGATCGTCGGCAGCGTC
	S518	5'-AATGATACGGCGACCACCGAGATCTACACCTATTAAGTCGTCGGCAGCGTC
	S520	5'-AATGATACGGCGACCACCGAGATCTACACAAGGCTATTCGTCGGCAGCGTC
	S521	5'-AATGATACGGCGACCACCGAGATCTACACGAGCCTTATCGTCGGCAGCGTC
	S522	5'-AATGATACGGCGACCACCGAGATCTACACTTATGCGATCGTCGGCAGCGTC
Index i7	N701	5'-CAAGCAGAAGACGGCATACGAGATTCGCCTTAGTCTCGTGGGCTCGG
	N702	5'-CAAGCAGAAGACGGCATACGAGATCTAGTACGGTCTCGTGGGCTCGG
	N703	5'-CAAGCAGAAGACGGCATACGAGATTTCTGCCTGTCTCGTGGGCTCGG
	N704	5'-CAAGCAGAAGACGGCATACGAGATGCTCAGGAGTCTCGTGGGCTCGG
	N705	5'-CAAGCAGAAGACGGCATACGAGATAGGAGTCCGTCTCGTGGGCTCGG
	N706	5'-CAAGCAGAAGACGGCATACGAGATCATGCCTAGTCTCGTGGGCTCGG
	N707	5'-CAAGCAGAAGACGGCATACGAGATGTAGAGAGGTCTCGTGGGCTCGG
	N710	5'-CAAGCAGAAGACGGCATACGAGATCAGCCTCGGTCTCGTGGGCTCGG
	N711	5'-CAAGCAGAAGACGGCATACGAGATTGCCTCTTGTCTCGTGGGCTCGG
	N712	5'-CAAGCAGAAGACGGCATACGAGATTCCTCTACGTCTCGTGGGCTCGG
	N714	5'-CAAGCAGAAGACGGCATACGAGATTCATGAGCGTCTCGTGGGCTCGG
	N715	5'-CAAGCAGAAGACGGCATACGAGATCCTGAGATGTCTCGTGGGCTCGG
	N716	5'-CAAGCAGAAGACGGCATACGAGATACTCGCTAGTCTCGTGGGCTCGG
	N718	5'-CAAGCAGAAGACGGCATACGAGATGGAGCTACGTCTCGTGGGCTCGG
	N719	5'-CAAGCAGAAGACGGCATACGAGATGCGTAGTAGTCTCGTGGGCTCGG
	N720	5'-CAAGCAGAAGACGGCATACGAGATCGGAGCCTGTCTCGTGGGCTCGG
	N721	5'-CAAGCAGAAGACGGCATACGAGATTACGCTGCGTCTCGTGGGCTCGG
	N722	5'-CAAGCAGAAGACGGCATACGAGATATGCGCAGGTCTCGTGGGCTCGG
	N723	5'-CAAGCAGAAGACGGCATACGAGATTAGCGCTC GTCTCGTGGGCTCGG
	N724	5'-CAAGCAGAAGACGGCATACGAGATACTGAGCGGTCTCGTGGGCTCGG
	N726	5'-CAAGCAGAAGACGGCATACGAGATCCTAAGACGTCTCGTGGGCTCGG
	N727	5'-CAAGCAGAAGACGGCATACGAGATCGATCAGTGTCTCGTGGGCTCGG
	N728	5'-CAAGCAGAAGACGGCATACGAGATTGCAGCTAGTCTCGTGGGCTCGG
	N729	5'-CAAGCAGAAGACGGCATACGAGATTCGACGTCGTCTCGTGGGCTCGG
